# Risk of Fracture With Dipeptidyl Peptidase-4 Inhibitors, Glucagon-like Peptide-1 Receptor Agonists, or Sodium-Glucose Cotransporter-2 Inhibitors in Patients With Type 2 Diabetes Mellitus: A Systematic Review and Network Meta-analysis Combining 177 Randomized Controlled Trials With a Median Follow-Up of 26 weeks

**DOI:** 10.3389/fphar.2022.825417

**Published:** 2022-07-01

**Authors:** Sanbao Chai, Fengqi Liu, Zhirong Yang, Shuqing Yu, Zuoxiang Liu, Qingqing Yang, Feng Sun

**Affiliations:** ^1^ Department of Endocrinology and Metabolism, Peking University International Hospital, Beijing, China; ^2^ Department of Epidemiology and Biostatistics, School of Public Health, Peking University Health Science Centre, Beijing, China; ^3^ Primary Care Unit, School of Clinical Medicine, University of Cambridge, Cambridge, United Kingdom

**Keywords:** DPP-4 inhibitors, GLP-1 receptor agonists, SGLT-2 inhibitors, fracture, diabetes mellitus

## Abstract

**Aim:** This study aims to investigate the association between the use of dipeptidyl peptidase-4 inhibitors (DPP-4i), glucagon-like peptide-1 receptor agonists (GLP-1 RAs), or sodium-glucose cotransporter-2 inhibitors (SGLT-2i) and the risk of fracture among patients with type 2 diabetes mellitus.

**Methods:** Medline, Embase, Cochrane Library, and Clinical-Trials.gov databases were searched for randomized controlled trials (RCTs). Network meta-analysis was performed for total fracture and a series of secondary outcomes.

**Results:** A total of 177 RCTs (*n* = 165,081) involving the risk of fracture were identified (a median follow-up of 26 weeks). DPP-4i, GLP-1 RAs, and SGLT-2i did not increase total fracture risk compared with insulin (odds ratio: 0.86, 95% confidence interval: 0.39–1.90; 1.05, 0.54–2.04; 0.88, and 0.39–1.97, respectively), metformin (1.41, 0.48–4.19; 1.72, 0.55–5.38; 1.44, 0.48–4.30), sulfonylureas (0.77, 0.50–1.20; 0.94, 0.55–1.62; 0.79, 0.48–1.31), thiazolidinediones (0.82, 0.27–2.44; 1.00, 0.32–3.10; 0.83, 0.27–2.57), α-glucosidase inhibitor (4.92, 0.23–103.83; 5.99, 0.28–130.37; 5.01, 0.23–107.48), and placebo (1.04, 0.84–1.29; 1.27, 0.88–1.83; 1.06, 0.81–1.39).

**Conclusions:** The use of DPP-4i, GLP-1 RAs, or SGLT-2i is unlikely to increase the risk of fracture among type 2 diabetes mellitus patients.

## 1 Introduction

There is an increased risk of fracture observed in both female and male patients with type 2 diabetes mellitus (T2DM) compared with non-diabetic individuals (Janghorbani et al., 2007; Formiga et al., 2020). Fracture in T2DM was significantly associated with severe disability, social burden, and reduction in quality of life (Hamann et al., 2012). The risk of fracture in T2DM patients may be attributed to reduced bone strength or poor bone quality, with varied effects of hypoglycemic drugs on bone metabolism. It is particularly important to determine whether hypoglycemic drugs can increase the risk of fracture.

A lot of research in this field has been carried out, and there are studies which reported the effects of different hypoglycemic drugs on fracture risk in T2DM (Lee et al., 2019; Salari-Moghaddam et al., 2019; Qian et al., 2020; Zhang et al., 2020). Dipeptidyl peptidase-4 inhibitors (DPP-4i) and the glucagon-like peptide-1 receptor agonists (GLP-1 RAs) have become widely used in T2DM patients as a novel class of blood glucose–lowering drugs with improved weight loss, low risk for hypoglycemia, and reduction in glycated hemoglobin (Drucker and Nauck, 2006; Ismail-Beigi, 2012; Cefalu et al., 2014). Sodium-glucose cotransporter-2 inhibitors (SGLT-2i) are another type of novel glucose-lowering agent and have also gained increasing use in recent years, which reduce plasma glucose concentrations by inhibiting proximal tubular reabsorption of glucose in the kidney (Davis et al., 2014). Though previous studies, including meta-analyses, have investigated the impact of DPP-4i, GLP-1 RAs, and SGLT-2i on the risk of fracture in patients with T2DM, their findings are not consistent (Su et al., 2015; Gamble et al., 2018; Adimadhyam et al., 2019; Cheng et al., 2019; Hidayat et al., 2019).

This network meta-analysis was performed to investigate the association between the use of DPP-4i, GLP-1 RAs, or SGLT-2i and the risk of fracture among patients with T2DM by synthesizing the data from all available randomized controlled trials (RCTs).

## 2 Participants and Methods

This study was conducted according to the Preferred Reporting Items for Systematic Reviews and Network Meta-Analyses (PRISMA-NMA) checklist.

### 2.1 Search Strategy

Medline, Embase, ClinicalTrials.gov, and the Cochrane Library were searched from inception to 7 September 2019. We used “Glucagon-Like Peptide-1 Receptor,” “Dipeptidyl-Peptidase IV Inhibitors,” and “Sodium-Glucose Cotransporter 2 Inhibitors” as keywords or MeSH terms, accompanied with relevant free words, to search these above databases. Details of search strategies are provided in Supplementary Appendix S1.

### 2.2 Study Selection

Only RCTs involving DPP-4i, GLP-1 RAs, or SGLT-2i compared with placebo or other antidiabetic agents [metformin (Met), insulin, sulfonylurea (SU), thiazolidinedione (TZD), and alpha-glucosidase inhibitor (AGI)] in patients with T2DM and reporting on any fracture as an outcome were included in this analysis (Supplementary Appendix S2). No other restrictions were applied to our eligibility criteria. The eligibility of studies was assessed independently by three reviewers (FL, SC, and FS), with any disagreement resolved by consensus.

### 2.3 Data Extraction and Quality Assessment

Data extraction was conducted by using the Aggregate Data Drug Information System (ADDIS version 1.16.5). Data extracted from eligible studies included trial information (first author, publication year, sample size, trial duration, types of interventions, and controls), baseline characteristics of patients (background therapy, duration of T2DM, age, baseline level of HbA1c, body weight, etc.), and results on bone fracture (including all fractures, upper limb fracture, lower limb fracture, hip fracture, etc.). Two investigators (FL and SC) extracted data independently, in duplicate.

The quality of studies was assessed according to the extracted information by Cochrane Collaboration’s tool for assessing risk of bias [including random sequence generation, allocation concealment, blinding of participants and personnel, blinding of outcome assessment, incomplete outcome data, selective reporting, and other sources of bias (i.e., company funding)] (Higgins et al., 2022). The Grading of Recommendations, Assessment, Development, and Evaluation (GRADE) system was used to rate the quality of evidence as high, moderate, low, or very low by taking into account the within-study limitations, imprecision, heterogeneity, indirectness, and publication bias for each outcome (University of Bern IoSaPM, 2017). We reported this study according to the Preferred Reporting Items for Systematic Reviews and Network Meta-Analyses (PRISMA-NMA) checklist (Hutton et al., 2015).

### 2.4 Data Analysis

#### 2.4.1 Methods for Direct Treatment Comparisons

Traditional pairwise meta-analysis was performed by using the DerSimonian–Laird random-effects model (DerSimonian and Laird, 1986). The odds ratio (OR) and 95% confidence interval (CI) for each outcome were calculated. I^2^ was used to assess the heterogeneity of direct treatment.

#### 2.4.2 Methods for Indirect and Mixed Comparisons

The primary outcome of this study is total fracture. In all studies included, some studies have reported the events of fractures in different body parts, for instance, spinal fracture, hip fracture, upper limb fracture, lower limb fracture, and other fractures. So network meta-analysis was also performed for a series of secondary outcomes of the specific fractures of our concern, including spinal fracture, hip fracture, upper limb fracture, lower limb fracture, and other fractures. We performed a frequentist random-effects network meta-analysis. OR with 95% CI was summarized and integrated into a network evidence body for each fracture outcome. We obtained the result of any pairwise comparison in the network evidence body through direct or indirect comparison. A node-splitting model (Dias et al., 2010) and a loop-specific approach (Higgins et al., 2012) were used to assess the inconsistency between direct and indirect treatment effects. A predictive interval plot that incorporates the extent of heterogeneity was used to evaluate the extent of uncertainty in the estimated effect size for the network meta-analysis. Uncertainty affected by heterogeneity was defined as a disagreement between the CIs of relative treatment effects and their predictive intervals. A series of box plots were drawn to compare whether there were significant differences in baseline age, HbA1c, duration of T2DM, sample size, and trial duration between different comparison pairs, so as to evaluate the transitivity assumption of this network body of evidence.

### 2.4.3 Publication Bias

The difference between the observed effect size and the comparison-specific summary effect for each study was calculated. Publication bias was evaluated according to whether the funnel diagram was symmetrical.

All analyses were conducted by using STATA Version.14.0 (pairwise meta-analysis, network meta-analysis, estimation of inconsistency and heterogeneity, and funnel plot) and R Version.4.1.1.

## 3 Results

### 3.1 Study Characteristics

**FIGURE 1:**
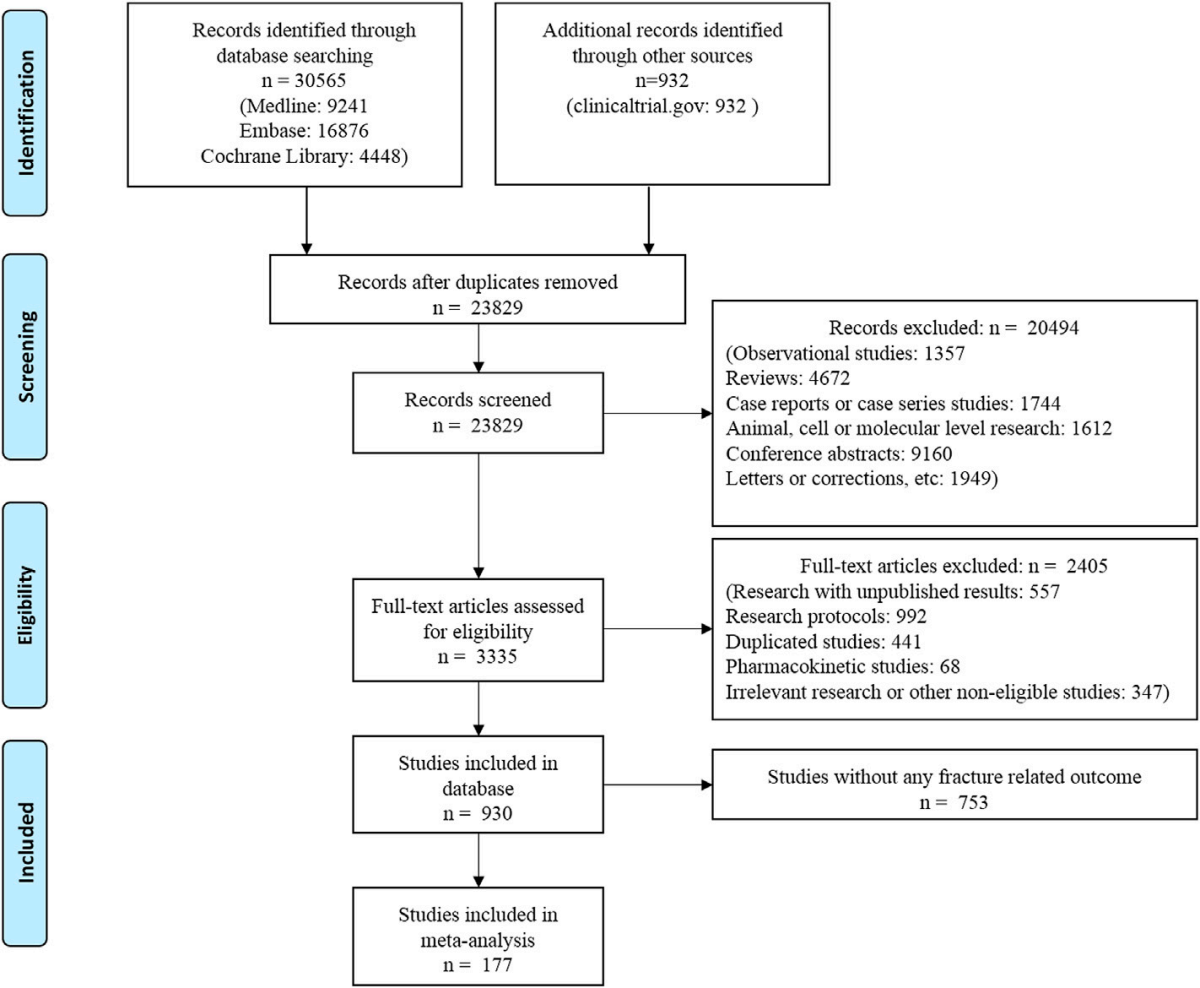
Flow chart of studies considered for inclusion.

The flow chart of the literature search is shown in Figure 1. Overall, 177 RCTs (*n* = 165,081 patients) met the eligibility criteria and were included in this network meta-analysis. Trial duration ranged from 12 to 384 weeks, with a median follow-up of 26 weeks (IQR: 24–54 weeks). The average age of the included patients was 57.68 years (SD: 5.08). The mean diabetes duration at baseline was 8.20 years (SD: 4.45) and the mean baseline HbA1c level was 8.14% (SD: 0.53%).

### 3.2 Evidence Network

**FIGURE 2:**
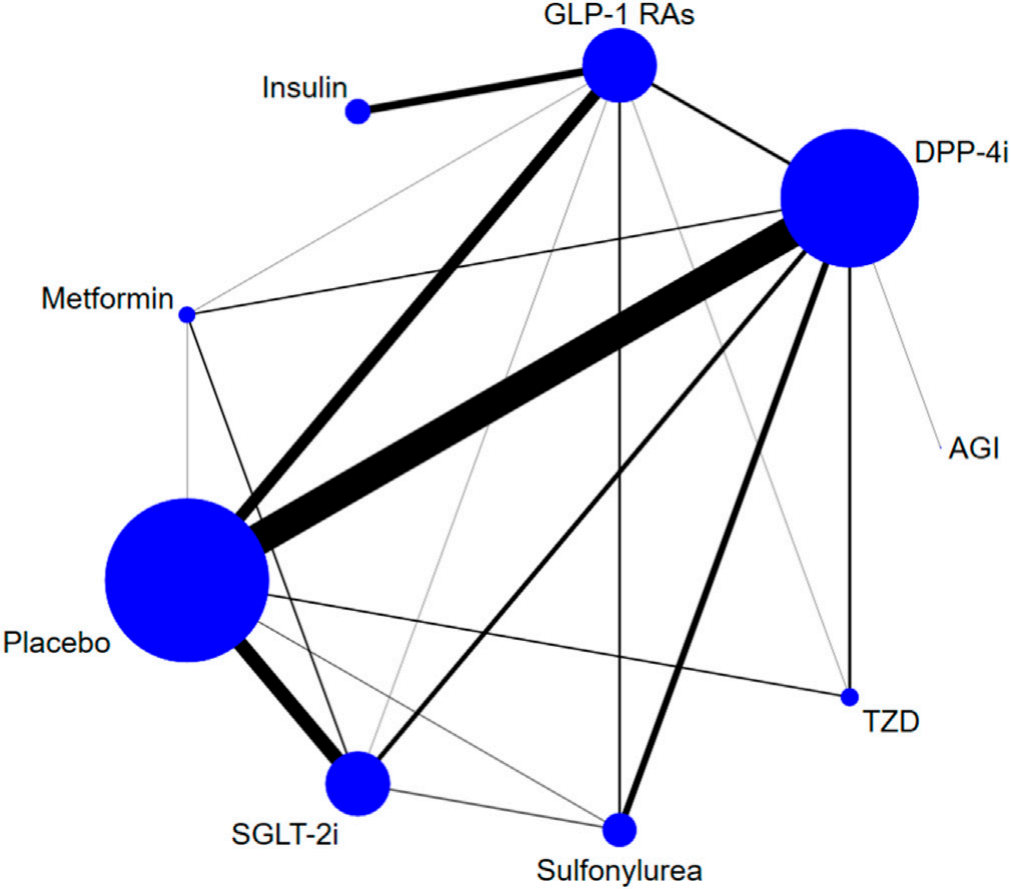
Evidence structure of DPP-4i–, GLP-1 RA–, and SGLT-2i–based therapies on risk of fracture. Note: The numbers along the link lines indicate the number of trials or pairs of trial arms. Lines connect the interventions that have been studied in head-to-head (direct) comparisons in the eligible randomized controlled trials. The width of the lines represents the cumulative number of randomized controlled trials for each pairwise comparison, and the size of every node is proportional to the number of randomized participants (sample size). DPP-4i: dipeptidyl peptidase-4 inhibitors; GLP-1 RAs: glucagon-like peptide-1 receptor agonists; TZD: thiazolidinedione; AGI: alpha-glucosidase inhibitor; SGLT-2i: sodium-glucose cotransporter-2 inhibitors.

Eight classes of treatments were analyzed, including DPP-4i (consisting of any drug among sitagliptin, vildagliptin, saxagliptin, linagliptin, alogliptin, teneligliptin, etc.), GLP-1 RAs (consisting of any drug among albiglutide, exenatide, lixisenatide, liraglutide, semaglutide, etc.), SGLT-2i (canagliflozin, dapagliflozin, empagliflozin, ipragliflozin, ertugliflozin, luseogliflozin, tofogliflozin, etc.), and five other classes of active antidiabetic agents (Met, insulin, SU, TZD, and AGI) and placebo. 166 trials (93.8%) were two-arm studies, 10 trials were three-arm studies, and one trial was a four-arm study (Figure 2). The box plots of baseline characteristics drawn according to the comparison pairs are shown in Supplementary Appendix S9. The patients’ baseline age (years), baseline HbA1c (%), baseline duration of T2DM (years), sample size, and trial duration (weeks) did not show significant differences among different comparison pairs, and there were few outliers, which indicated that the transitivity assumption of the network evidence body was established (Supplementary Appendix S3).

### 3.3 Risk of Bias

For the total 177 studies included in this analysis, majority of the studies were determined to be at “low risk” of bias in random sequence generation (151/177, 85.3%), blinding of participants and personnel (142/177, 80.2%), and blinding of outcome assessment (140/177, 79.1%). Some studies were judged as “low risk” of bias in allocation concealment (97/177, 54.8%), complete outcome data (65/177, 36.7%), and selective reporting (65/177, 36.7%). Only a few studies were rated as “high risk” of bias in the above items. 46.3% of the studies were funded by enterprises. Overall, the risk of bias across the evidence network was relatively low (Supplementary Appendix S4).

### 3.4 Direct Treatment Comparisons

We conducted a series of traditional paired meta-analysis on all interventions that have a direct comparison between these two for total fracture. The comparison includes placebo vs. DPP-4i, DPP-4i vs. AGI, SGLT-2i vs. GLP-1 RAs, SGLT-2i vs. metformin, SGLT-2i vs. placebo, TZD vs. DPP-4i, GLP-1 RAs vs. metformin, GLP-1 RAs vs. placebo, TZD vs. placebo, GLP-1 RAs vs. DPP-4i, GLP-1 RAs vs. insulin, GLP-1 RAs vs. sulfonylurea, GLP-1 RAs vs. TZD, sulfonylurea vs. placebo, DPP-4i vs. metformin, DPP-4i vs. SGLT-2i, DPP-4i vs. sulfonylurea, metformin vs. placebo, and SGLT-2i vs. sulfonylurea. The results of all the above comparisons show that there is no significant statistical difference between the two compared; that is, there is no significant difference in the risk of fracture in the above comparisons (Supplementary Appendix S5).

### 3.5 Network Meta-Analysis of DPP-4i, GLP-1 RAs, and SGLT-2i on Total Fracture Risk and Secondary Outcomes

**FIGURE 3:**
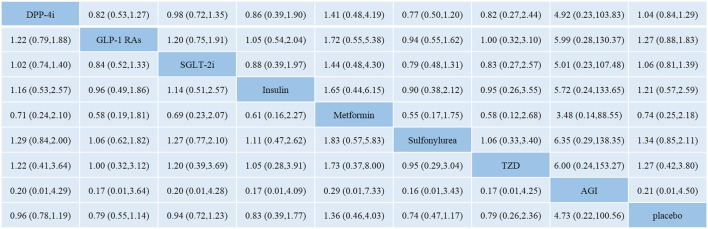
Odds ratio with 95% CI of network meta-analysis for risk of fracture. Note: Results of direct comparisons were listed in the upper triangle, and the estimation was calculated as the row-defining treatment compared with the column-defining treatment. Results of network meta-analysis were listed in the lower triangle, and the estimation was calculated as the column-defining treatment compared with the row-defining treatment. NA: not available. DPP-4i: dipeptidyl peptidase-4 inhibitors; GLP-1 RAs: glucagon-like peptide-1 receptor agonists; SGLT-2i: sodium-glucose cotransporter-2 inhibitors; TZD: thiazolidinedione; AGI: alpha-glucosidase inhibitor.

Figure 3 shows the network meta-analysis results of the comparative effect of DPP-4i, GLP-1 RAs, SGLT-2i, other antidiabetic agents, and placebo on total fracture risk. DPP-4i did not increase total fracture risk compared with insulin (OR: 0.86, 95% CI: 0.39–1.90), Met (OR: 1.41, 95% CI: 0.48–4.19), SU (OR: 0.77, 95% CI: 0.50–1.20), TZD (OR: 0.82, 95% CI: 0.27–2.44), AGI (OR: 4.92, 95% CI: 0.23–103.83), and placebo (OR: 1.04, 95% CI: 0.84–1.29), respectively.

GLP-1 RAs did not increase fracture risk compared with insulin (OR: 1.05, 95% CI: 0.54–2.04), Met (OR: 1.72, 95% CI: 0.55–5.38), SU (OR: 0.94, 95% CI: 0.55–1.62), TZD (OR: 1.00, 95% CI: 0.32–3.10), AGI (OR: 5.99, 95% CI: 0.28–130.37), and placebo (OR: 1.27, 95% CI: 0.88–1.83), respectively.

SGLT-2i did not increase fracture risk compared with insulin (OR: 0.88, 95% CI: 0.39–1.97), Met (OR: 1.44, 95% CI: 0.48–4.30), SU (OR: 0.79, 95% CI: 0.48–1.31), TZD (OR: 0.83, 95% CI: 0.27–2.57), AGI (OR: 5.01, 95% CI: 0.23–107.48), and placebo (OR: 1.06, 95% CI: 0.81–1.39), respectively.

Secondary outcomes based on fracture of different parts indicated that DPP-4i, GLP-1 RAs, and SGLT-2i did not increase the risk of fracture, respectively (Supplementary Appendix S6).

### 3.6 Inconsistency and Heterogeneity Test

The result of local inconsistency about fracture risk showed that all loops were consistent according to the CIs. The test for inconsistency using the node-splitting model revealed no significant difference about the total fracture between direct and indirect comparisons (global inconsistency, *p* = 0.97). The predictive interval plot showed that there was little heterogeneity in this study. Summary estimations of network meta-analysis were relatively robust (Supplementary Appendix S7). The fracture outcomes in secondary outcomes were also tested for inconsistency and heterogeneity, and have not been tested for significant heterogeneities or inconsistencies either.

### 3.7 Publication Bias

Funnel plots were shown in Supplementary Appendix S8. For total fracture, scatters in the funnel plot were almost symmetrical, visually. But the linear regression line was close to horizontal, indicating that the publication bias in the result of total fracture between small and large studies was relatively high. For other five secondary outcomes, the scatters in the funnel plots were almost symmetrical, visually.

### 3.8 Quality of Evidence

The GRADE process was completed using CINeMA software (http://cinema. ispm.ch/). The quality of most studies was moderate. With concern of within-study bias and imprecision, the quality of evidence in the total fracture was rated as moderate (Supplementary Appendix S9).

## 4 Discussion

In the study, we analyzed 177 eligible RCTs, including 165,081 patients. Our network meta-analysis showed that 1) there was no evidence to indicate an increased risk of fracture associated with DPP-4i–, GLP-1 RA–, and SGLT-2i–based therapies in patients with T2DM compared with other antidiabetic agents or placebo; 2) secondary outcomes based on fracture of different parts indicated that DPP-4i, GLP-1 RAs, and SGLT-2i did not increase the risk of fracture, respectively.

In agreement with our findings, a meta-analysis of observational studies did not support an association between the use of DPP-4i, GLP-1 RAs, or SGLT-2i and the risk of fracture (Hidayat et al., 2019). A meta-analysis of 51 RCTs reported that there was no significant association between DPP-4 inhibitor use and the incidence of fractures, when DPP-4 inhibitor is compared with placebo or an active comparator (Mamza et al., 2016). Fu et al. (2016) reported that DPP-4 inhibitor use does not modify the risk of bone fracture compared with placebo or other antidiabetic medications in patients with T2DM (RR = 0.95; 95% CI: 0.83–1.10). A population-based cohort study showed that the use of GLP-1 RAs versus other anti-hyperglycemic drugs was not associated with fracture risk (Driessen et al., 2015a). Multiple meta-analyses have indicated that SGLT-2i does not increase the risk of bone fracture compared with placebo in patients with T2DM (Ruanpeng et al., 2017; Azharuddin et al., 2018; Cheng et al., 2019). Toulis et al. (Toulis et al., 2018) conducted a retrospective cohort study by using Health Improvement Network data and reported that patients initiating dapagliflozin did not have an elevated risk for fractures compared with patients initiating any other antidiabetic medication [HR 0.89 (95% CI: 0.66–1.20)]. In this population-based new-user cohort study using data from two large US healthcare databases, canagliflozin use was not associated with an increased risk of fracture compared to GLP-1 agonists (Fralick et al., 2019).

However, contrasting evidence emerged from a meta-analysis suggesting exenatide treatment was associated with an elevated risk of incident fractures (MHOR = 2.09; 95% CI: 1.03–4.21) compared to placebo or other active drugs (Su et al., 2015). The reasons for the inconsistency of the research conclusions may be related to the low number of fracture cases in this meta-analysis and the durations of trials. There are also observational studies showing that the use of SGLT-2i can increase the risk of fracture. A retrospective cohort study using the Truven Health Market Scan (2009–2015) database observed an 82% increase in risk of fractures within the first 2 weeks of treatment with SGLT-2i, with 95% CI of 0.99–3.32 (Adimadhyam et al., 2019). In the Canagliflozin Cardiovascular Assessment Study (CANVAS) Program, a significant increase in fractures was seen with canagliflozin (4.0%) vs. placebo (2.6%), and the incidence of fractures was higher with canagliflozin (2.7%) vs. noncanagliflozin (1.9%) in the overall population (Watts et al., 2016). The inconsistent results may be related to the use time of SGLT-2i and the presence or absence of TZD, which is known to have an increased risk of fractures (Meier et al., 2008; Loke et al., 2009).

As a robust predictor of fracture risk, bone mineral density (BMD) is a surrogate reflecting bone strength. While (Hidayat et al., 2019) reported that basal DPP-4 activity was not significantly associated with BMD of the hip, lumbar spine or total body, or incident hip fractures in elderly community-dwelling men and women. In a cohort of elderly community-dwelling adults, plasma DPP-4 activity was not associated with BMD or incident hip fractures (Carbone et al., 2017). Iepsen et al. (2015) showed the use of liraglutide increased bone formation and prevented bone loss after weight loss in obese women. Dapagliflozin had no effect on bone formation and resorption or BMD in both male and post-menopausal female patients (Ljunggren et al., 2012). While the relationship between hypoglycemic drugs and BMD may reflect the effects of drugs on fracture risk, our systematic review could not provide evidence on BMD as all RCTs included in this review did not conduct BMD test for diabetic patients.

Previous studies have reported fracture-prone sites in diabetic patients. A systematic review (Janghorbani et al., 2007) indicated that type 2 diabetes was associated with an increased risk of hip fracture in both men and women, but not with fractures of the distal forearm, ankle, proximal humerus, or vertebra. A nested case-control study among patients with type 2 diabetes suggested that the use of SGLT-2i and other antidiabetic drug classes was not associated with an increased risk of fractures of the upper or lower limbs compared to use of DPP-4 inhibitors (Schmedt et al., 2019). Consistent with most of these findings, our analysis based on different parts of the body did not find that the use of DPP-4i, GLP-1 RAs, and SGLT-2i increased the risk of fracture. The results of this study suggested that TZD did not increase the risk of fracture. Because this study mainly evaluated the fracture risk of three new hypoglycemic drugs (SGLT-2 inhibitors, GLP-1 RAs, and DPP-4 inhibitors), the condition for searching the literature is that at least one arm is any one of these three drugs. The study on the comparison of TZD and other hypoglycemic drugs is not the outcome of this study, so it is not included in this study, especially the RCT study used to evaluate the safety related to the comparison of TZD and placebo. Owing to the purpose of this study and the characteristics of the included literature, the results of TZD compared with other hypoglycemic drugs are mainly indirect comparisons. CIs are generally wide, and there may be an outcome that TZD does not increase the risk of fracture.

Recently, studies on GLP-1 RAs, DPP-4i, and SGLT-2i in fracture risk of type 2 diabetes patients showed that gender differences may not be enough to play a decisive role in the increase of fracture risk in T2DM (Driessen et al., 2015b; Hou et al., 2018; Rådholm et al., 2020; Davie et al., 2021). Therefore, this study did not analyze gender factors.

Our study has several strengths. First, the predictive interval plot showed that there was little heterogeneity in this study. Second, in addition to assessing the overall risk of fractures, our study also assessed the risk of fractures in different parts of the body.

This study also has some limitations. First, none of the RCTs included were tested for BMD and bone metabolism indexes, which are closely related to the risk of fracture. Second, some CIs in results are particularly wide, for example, the result range of AGI compared with placebo. The reason is that both interventions are used as the control group in this study, and the comparison of results between AGI and placebo are based on indirect comparison. Third, this study regards all the same types of hypoglycemic drug (regardless of the dose or specific drug) as the same intervention and has not considered the effects of specific drugs and their doses on the outcomes. Fourth, most of the included RCTs were conducted in Western countries, but whether they are suitable for other regions, such as Asian populations, remains to be discussed. Fifth, the literature retrieval time of this study is up to 2019, so the results of the latest research could not be included in time.

In conclusion, our meta-analysis suggests that the use of DPP-4i, GLP-1 RAs, and SGLT-2i is not associated with an increased risk of fracture in patients with T2DM. In addition, more RCTs are required to investigate the number of fractures with the use of DPP-4i, GLP-1 RAs, and SGLT-2i, as a primary endpoint, rather than an adverse event.

## Conclusion

Dipeptidyl peptidase-4 inhibitors (DPP-4i) and the glucagon-like peptide-1 receptor agonists (GLP-1 RAs) have become widely used in T2DM patients as a novel class of blood glucose–lowering drugs with improved weight loss, low risk for hypoglycemia, and reduction in glycated hemoglobin. Sodium-glucose cotransporter-2 inhibitors (SGLT-2i) are another type of novel glucose-lowering agents and have also gained increasing use in recent years, which reduce plasma glucose concentrations by inhibiting proximal tubular reabsorption of glucose in the kidney.

There are two new findings in the study. First, the use of DPP-4i, GLP-1 RAs, or SGLT-2i is unlikely to increase the overall risk of fracture among type 2 diabetes mellitus patients. Second, secondary outcomes based on fracture of different parts indicated that DPP-4i, GLP-1 RAs, and SGLT-2i did not increase the risk of fracture, respectively.

Our results offer the best available evidence of the impact of DPP-4i, GLP-1 RAs, or SGLT-2i on fracture risk based on randomized controlled trials. Therefore, more evidence is provided for the rational use of these drugs in clinical trials.

## Data Availability

The raw data supporting the conclusions of this article will be made available by the authors without undue reservation.
