# Supplementary material for: Risk of Fracture With Dipeptidyl Peptidase-4 Inhibitors, Glucagon-like Peptide-1 Receptor Agonists, or Sodium-Glucose Cotransporter-2 Inhibitors in Patients With Type 2 Diabetes Mellitus: A Systematic Review and Network Meta-analysis Combining 177 Randomized Controlled Trials With a Median Follow-Up of 26 weeks
Source: Front Pharmacol. 2022 Jul 1;13:825417. doi: 10.3389/fphar.2022.825417 (PMC9285982; doi:10.3389/fphar.2022.825417)
Supplement: Supplementary file 1 [file DataSheet6.docx]

Supplementary appendix 7 Evaluation of the inconsistency and heterogeneity for all closed loops

in the network

**Global inconsistency test**

In the process of network meta-analysis by STATA Version.14.0, we used the total fracture data to fit the inconsistent model, the result shows: Chi-square=10.01, P=0.9680 > 0.05, so the overall inconsistency of this data was considered to be insignificant, that is, consistent model can be chosen for analysis.

**Local inconsistency test**

We test Local inconsistency by node-splitting test, the result was as shown in Table 1. All P values were greater than 0.05, and the local inconsistency was not significant.

Table 1 The result of node-splitting test

| Side | Direct | | Indirect | | Difference | | |
| --- | --- | --- | --- | --- | --- | --- | --- |
|  | Coef. | Std. Err. | Coef. | Std. Err. | Coef. | Std. Err. | P>\|z\| |
| A B * | -1.598884 | 1.558891 | 0.1591882 | 26.29808 | -1.758072 | 26.34425 | 0.947 |
| B C | -0.0795706 | 0.4864503 | -0.2203933 | 0.2462001 | 0.1408227 | 0.5434338 | 0.796 |
| B E | -0.0219658 | 0.8215412 | 0.6564787 | 0.7520952 | -0.6784444 | 1.113871 | 0.542 |
| B F | 0.0839273 | 0.4954104 | -0.0300787 | 0.1705226 | 0.1140061 | 0.5245606 | 0.828 |
| B G | -0.3331494 | 0.2604409 | -0.0359033 | 0.4356381 | -0.2972462 | 0.5077431 | 0.558 |
| B H | -0.2896966 | 0.6729517 | -0.0123011 | 0.9554214 | -0.2773954 | 1.1521 | 0.81 |
| B I | 0.0669423 | 0.1234997 | -0.1054915 | 0.3173231 | 0.1724337 | 0.3466494 | 0.619 |
| C D * | 0.0459283 | 0.3392778 | 3.837173 | 173.4811 | -3.791245 | 173.4814 | 0.983 |
| C E | -0.402678 | 1.640235 | 0.6787881 | 0.6206992 | -1.081466 | 1.75375 | 0.537 |
| C F | -1.115896 | 1.641005 | 0.2056442 | 0.2381293 | -1.32154 | 1.658193 | 0.425 |
| C G | 0.1130114 | 0.4553176 | -0.1676666 | 0.349903 | 0.280678 | 0.5763769 | 0.626 |
| C H | -0.0219307 | 1.166628 | 0.0017264 | 0.6672269 | -0.0236571 | 1.343957 | 0.986 |
| C I | 0.3472824 | 0.1792859 | -0.0402215 | 0.363013 | 0.3875039 | 0.4056553 | 0.339 |
| E F | -1.038913 | 0.8908883 | 0.0965549 | 0.7336 | -1.135468 | 1.17061 | 0.332 |
| E I | -1.515657 | 1.597759 | -0.1188464 | 0.6022245 | -1.396811 | 1.732332 | 0.42 |
| F G | 0.4948189 | 0.7266976 | -0.3429515 | 0.2755359 | 0.8377704 | 0.7771828 | 0.281 |
| F I | 0.0308126 | 0.1427218 | 0.334088 | 0.4418297 | -0.3032754 | 0.4628735 | 0.512 |
| G I | 0.6576462 | 0.8943837 | 0.2673375 | 0.2411947 | 0.3903087 | 0.9315708 | 0.675 |
| H I | -0.1899864 | 0.8786051 | 0.5988325 | 0.7974583 | -0.7888189 | 1.245844 | 0.527 |

( A : AGI; B: DPP-4i; C: GLP-1 RAs; D: Insulin; E: Metformin; F: SGLT-2i; G: Sulfonylurea; H: TZD; I: placebo)

* all the evidence about these contrasts comes from the trials which directly compare them.

**Loop inconsistency test**

As can be seen in Figure 1, all confidence intervals includes 0, and the inconsistency based on loop was not significant.


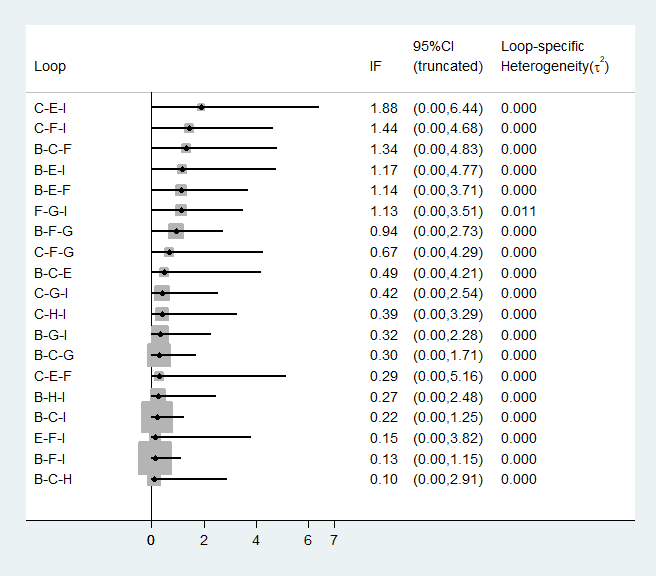


*( A : AGI; B: DPP-4i; C: GLP-1 RAs; D: Insulin; E: Metformin; F: SGLT-2i; G: Sulfonylurea; H: TZD; I: placebo)

Figure 1 The result of loop inconsistency test

**Heterogeneity test**

As can be seen in Figure 2, there are a total of 9 interventions involved in the study, among which the results of comparing any two interventions, there is no significant difference between 95% CIs and the predictive intervals, so the heterogeneity in the study can be considered not significant.


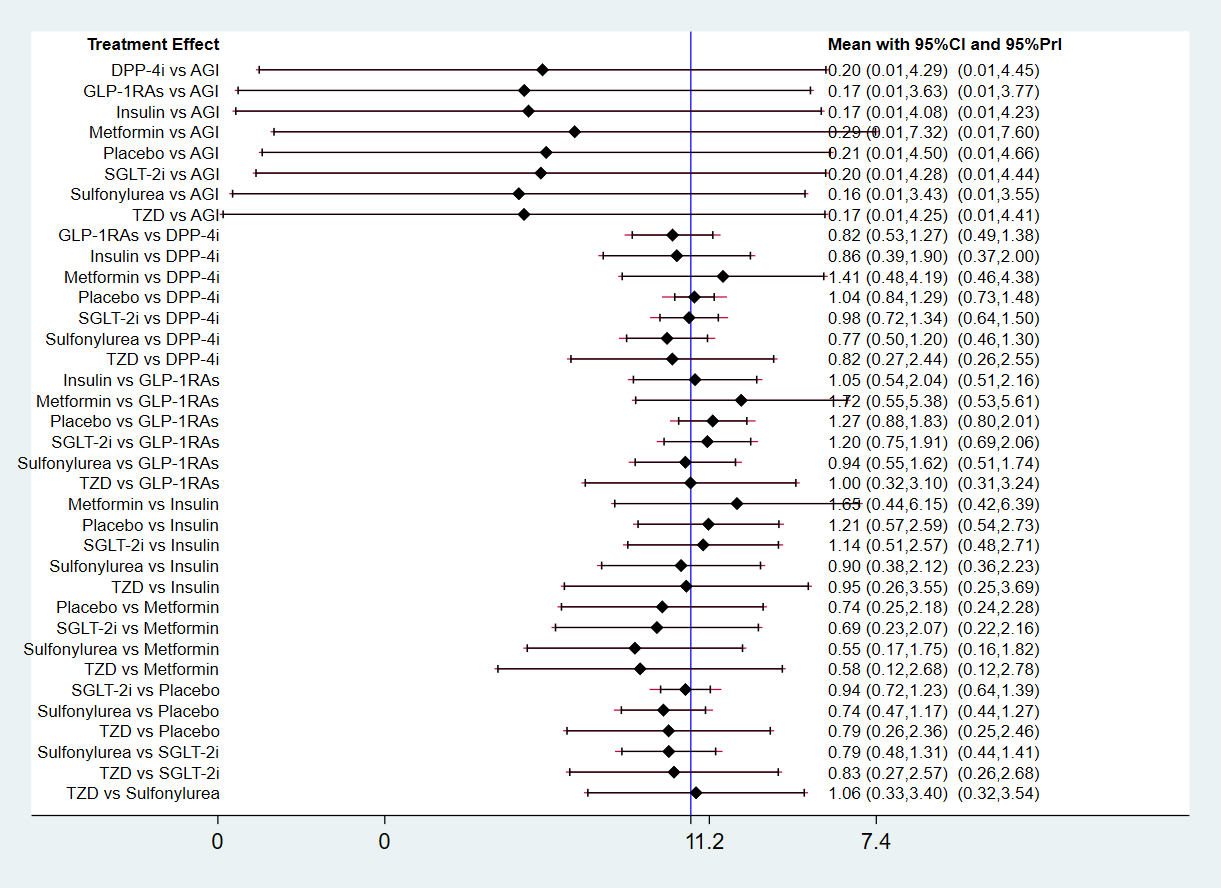


Figure 2 Forest plot with predictive interval
