# Supplementary material for: Risk of Fracture With Dipeptidyl Peptidase-4 Inhibitors, Glucagon-like Peptide-1 Receptor Agonists, or Sodium-Glucose Cotransporter-2 Inhibitors in Patients With Type 2 Diabetes Mellitus: A Systematic Review and Network Meta-analysis Combining 177 Randomized Controlled Trials With a Median Follow-Up of 26 weeks
Source: Front Pharmacol. 2022 Jul 1;13:825417. doi: 10.3389/fphar.2022.825417 (PMC9285982; doi:10.3389/fphar.2022.825417)
Supplement: Supplementary file 2 [file DataSheet3.docx]

Supplementary appendix 3 Transitivity test


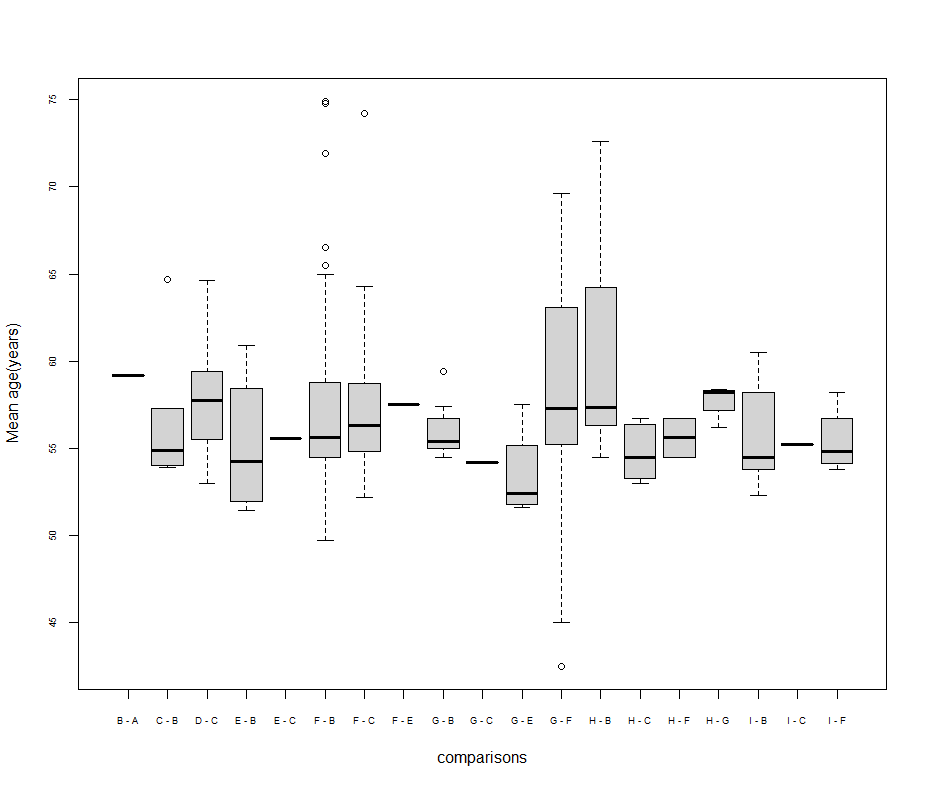


Figure 1 Box plot for baseline age by comparisons (years)


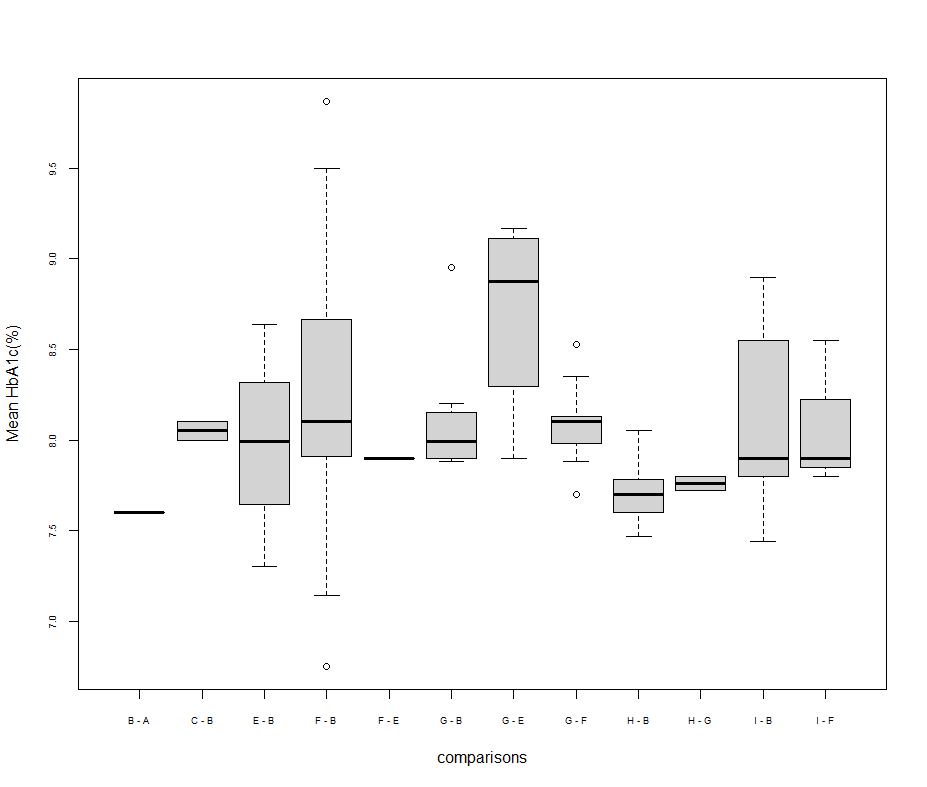


Figure 2 Box plot for baseline HbA1c by comparisons (%)


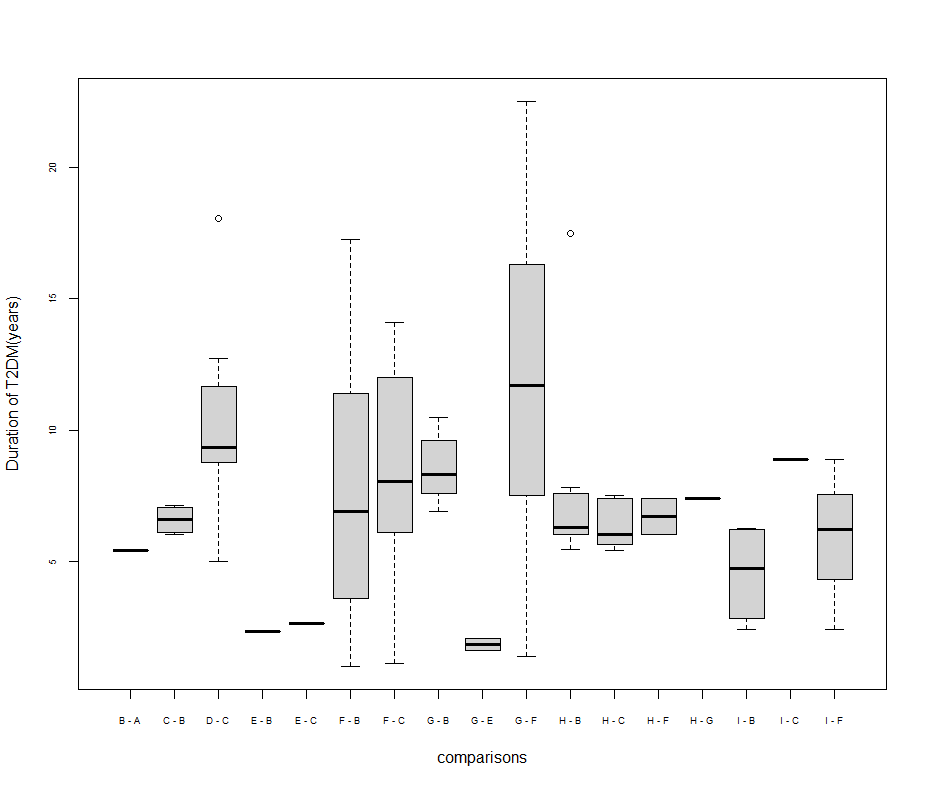


Figure 3 Box plot for baseline duration of T2DM by comparisons (years)


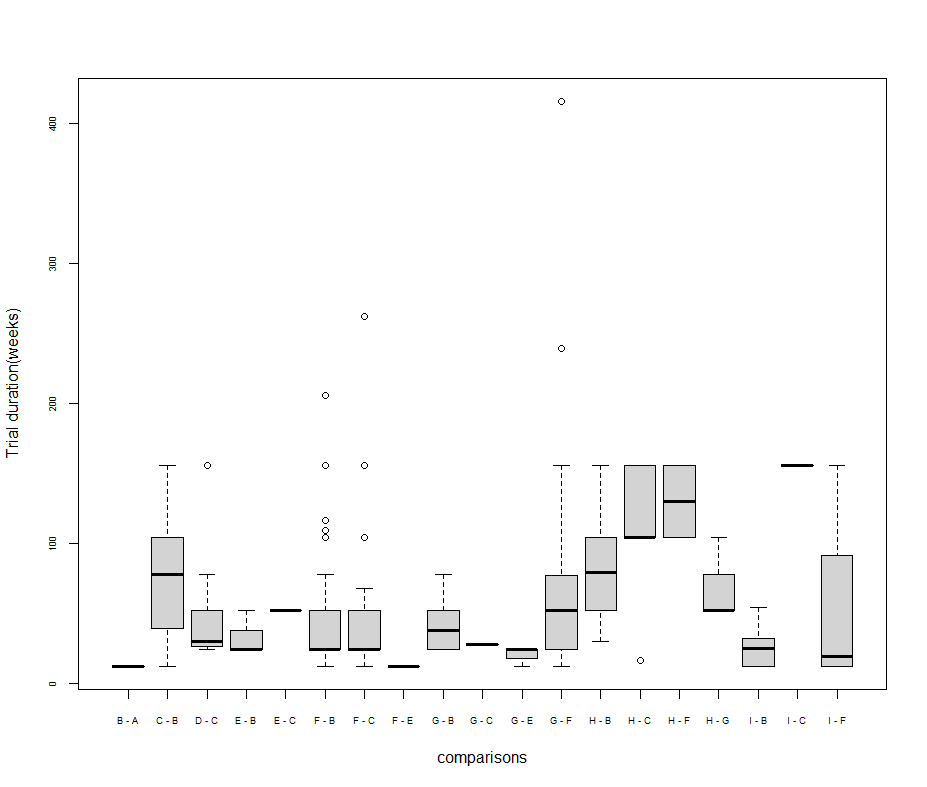


Figure 4 Box plot for trial duration by comparisons (weeks)


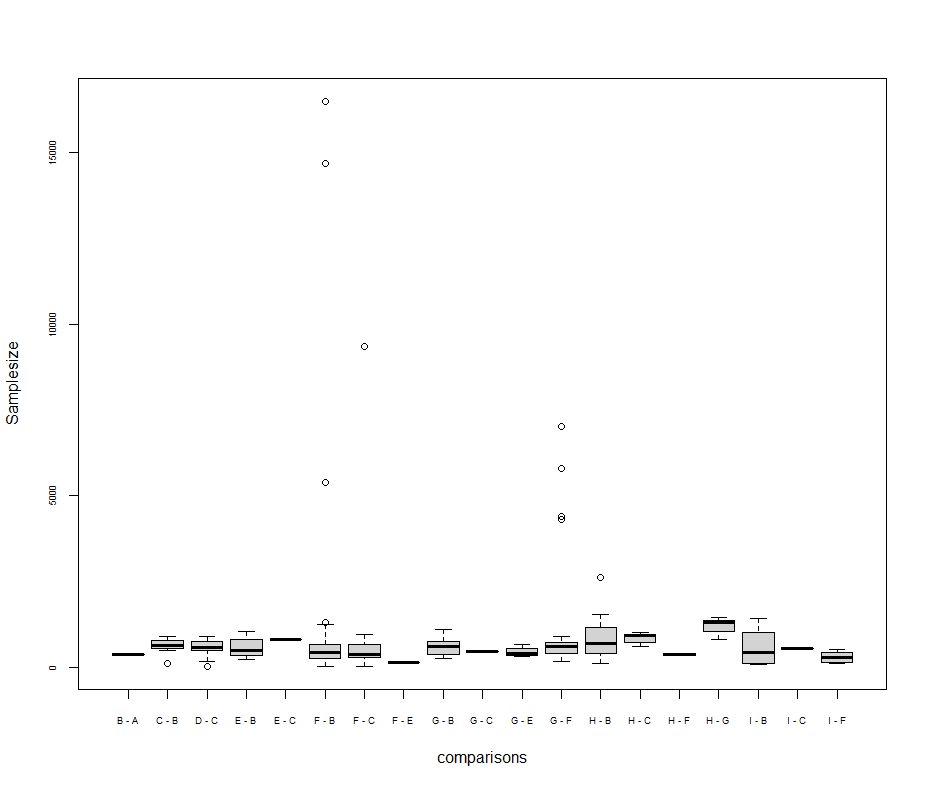


Figure 5 Box plot for sample size by comparisons
