# Supplementary material for: Risk of Fracture With Dipeptidyl Peptidase-4 Inhibitors, Glucagon-like Peptide-1 Receptor Agonists, or Sodium-Glucose Cotransporter-2 Inhibitors in Patients With Type 2 Diabetes Mellitus: A Systematic Review and Network Meta-analysis Combining 177 Randomized Controlled Trials With a Median Follow-Up of 26 weeks
Source: Front Pharmacol. 2022 Jul 1;13:825417. doi: 10.3389/fphar.2022.825417 (PMC9285982; doi:10.3389/fphar.2022.825417)
Supplement: Supplementary file 3 [file DataSheet1.doc]

Supplementary appendix 1 Search strategy

**1. Medline：**

#1 *Glucagon-Like Peptide-1 Receptor/ or glucagon-like peptide-1 agonists.mp. or *Glucagon-Like Peptide 1/

#2 "glucagon like peptide*".ab,ti.

#3 Receptors, Glucagon/ag [Agonists]

#4 exenatide.af.

#5 liraglutide.af.

#6 albiglutide.af.

#7 taspoglutide.af.

#8 lixisenatide.af.

#9 dulaglutide.af.

#10 semaglutide.af.

#11 Byetta.af.

#12 Bydureon.af.

#13 Victoza.af.

#14 Lyxumia.af.

#15 Adlyxin.af.

#16 Tanzeum.af.

#17 Eperzan.af.

#18 Trulicity.af.

#19 ZP10A peptide*.af.

#20 AVE 0010.af.

#21 Ozempic.af.

#22 GLP 1 Receptor Agonist*.af.

#23 GLP 1 RA*.af.

#24 GLP 1RA*.af.

#25 #1 or #2 or #3 or # 4 or #5 or #6 or #7 or #8 or #9 or #10 or #11 or #12 or #13 or #14 or #15 or #16 or #17 or #18 or #19 or #20 or #21 or #22 or #23 or #24

#26 Dipeptidyl Peptidase 4 inhibitor.mp. or exp *Dipeptidyl-Peptidase IV Inhibitors/

#27 dipeptidyl peptidase IV inhibit*.af.

#28 Dipeptidyl Peptidase 4 Inhibit*.af.

#29 DPP 4 inhibit*.af.

#30 DPP4 inhibit*.af.

#31 DPP4i.af.

#32 DPP IV inhibit*.af.

#33 DPPIV inhibit*.af.

#34 sitagliptin.af.

#35 Januvia.af.

#36 Janumet.af.

#37 Juvisync.af.

#38 vildagliptin.af.

#39 Galvus.af.

#40 Eucreas.af.

#41 Galvus Met.af.

#42 saxagliptin.af.

#43 Onglyza.af.

#44 Kombiglyze XR.af.

#45 Qtern.af.

#46 alogliptin.af.

#47 Nesina.af.

#48 Oseni.af.

#49 Kazano.af.

#50 Vipidia.af.

#51 Vipdoment.af.

#52 linagliptin.af.

#53 Trajenta.af.

#54 gemigliptin.af.

#55 Gemiglo.af.

#56 anagliptin.af.

#57 Beskoa.af.

#58 teneligliptin.af.

#59 Tenelia.af.

#60 Diabeglipt.af.

#61 Elant.af.

#62 Glucal.af.

#63 Teneglucon.af.

#64 Veriglip.af.

#65 Glipten.af.

#66 Trelagliptin.af.

#67 PF 734200.af.

#68 retagliptin.af.

#69 Melogliptin.af.

#70 evogliptin.af.

#71 Suganon.af.

#72 Carmegliptin.af.

#73 LC15 0444.af.

#74 DA-1229.af.

#75 omarigliptin.af.

#76 gliptin*.af.

#77 dutogliptin.af.

#78 #26 or #27 or #28 or #29 or #30 or #31 or #32 or #33 or #34 or #35 or #36 or #37 or #38 or #39 or #40 or #41 or #42 or #43 or #44 or #45 or #46 or #47 or #48 or #49 or #50 or #51 or #52 or #53 or #54 or #55 or #56 or #57 or #58 or #59 or #60 or #61 or #62 or #63 or #64 or #65 or #66 or #67 or #68 or #69 or #70 or #71 or #72 or #73 or #74 or #75 or #76 or #77

#79 sodium-glucose cotransporter 2 inhibitor.mp. or exp *sodium-glucose transporter 2 inhibitors/

#80 sodium-glucose cotransporter 2*.ab,ti.

#81 Sodium-Glucose Transporter 2 inhibit*.af.

#82 Sodium-Glucose cotransporter 2 Inhibit*.af.

#83 Sodium-dependent glucose transporter 2 inhibit*.af.

#84 Sodium glucose transporter 2 inhibit*.af.

#85 SGLT2 inhibit*.af.

#86 SGLT 2 inhibit*.af.

#87 SGLTⅡ inhibit*.af.

#88 SGLT Ⅱ inhibit*.af.

#89 SGLT2i.af.

#90 Empagliflozin.af.

#91 Invokana.af.

#92 Canagliflozin.af.

#93 Forxiga.af.

#94 Dapagliflozin.af.

#95 Rongliflozin.af.

#96 Ipragliflozin.af.

#97 Luseogliflozin.af.

#98 Tofogliflozin.af.

#99 Ertugliflozin.af.

#100 Glyxambi.af.

#101 Jardiance.af.

#102 Bexagliflozin.af.

#103 Sotagliflozin.af.

#104 Licogliflozin.af.

#105 Sergliflozin.af.

#106 Henagliflozin.af.

#107 Remogliflozin.af.

#108 JNJ28431754.af.

#109 TA7284.af.

#110 BI10773.af.

#111 SAR439954.af.

#112 LX4211.af.

#113 EGT0001442.af.

#114 ASP1941.af.

#115 #79 or #80 or #81 or #82 or #83 or #84 or #85 or #86 or #87 or #88 or #89 or #90 or #91 or #92 or #93 or #94 or #95 or #96 or #97 or #98 or #99 or #100 or #101 or #102 or #103 or #104 or #105 or #106 or #107 or #108 or #109 or #110 or #111 or #112 or #113 or #114

#116 #25 or #78 or #115

#117 (clinical trial or controlled clinical trial or randomized controlled trial).pt.

#118 clinical trials.mp. or exp *clinical trial/

#119 clinical trials as topic/ or controlled clinical trials as topic/ or randomized controlled trials as topic/

#120 random*.ti,ab.

#121 clinical trial*.ti,ab.

#122 controlled trial*.ti,ab.

#123 comparison group*.ti,ab.

#124 control group*.ti,ab.

#125 trial*.ti,ab.

#126 placebo*.ti,ab.

#127 registries/

#128 registries.ti,ab.

#129 trial$1 register.ti.

#130 trial$1 registers.ti.

#131 #117 or #118 or #119 or #120 or #121 or #122 or #123 or #124 or #125 or #126 or #127 or #128 or #129 or #130

#132 #25 and #131

#133 #78 and #131

#134 #115 and #131

#136 limit #132 to humans

#137 limit #132 to animals

#138 #137 not #136

#139 #132 not #138

#140 limit #133 to humans

#141 limit #133 to animals

#142 #141 not #140

#143 #133 not #142

#144 limit #134 to humans

#145 limit #134 to animals

#146 #145 not #144

#147 #134 not #146

#148 #139 or #143 or #147

#149 meta analysis.pt.

#150 meta-Analysis as Topic/

#151 meta analy*.ti.

#152 metaanaly*.ti.

#153 #149 or #150 or #151 or #152

#154 #25 and #153

#155 #78 and #153

#156 #115 and #153

#157 limit #154 to humans

#158 limit #154 to animals

#159 #158 not #157

#160 #154 not #159

#161 limit #155 to humans

#162 limit #155 to animals

#163 #162 not #161

#164 #155 not #163

#165 limit #156 to humans

#166 limit #156 to animals

#167 #166 not #165

#168 #156 not #167

#169 #139 not #160

#170 #143 not #164

#171 #147 not #168

#172 #169 or #170 or #171

**2. Embase：**

#1 'glucagon like peptide'/exp

#2 'glucagon like peptide 1'/exp

#3 'glp-1 receptor agonists'

#4 'glucagon like peptide 1 receptor agonists'

#5 'glucagon receptor'/exp

#6 'glucagon-like peptide-1 agonists'

#7 'glp-1 receptor agonist'

#8 'glucagon like peptide 1 receptor agonist'

#9 'glp-1 agonist'

#10 'glp-1 agonists'

#11 'glp-1 ra*'

#12 'exenatide'/exp

#13 'liraglutide'/exp

#14 'albiglutide'/exp

#15 'taspoglutide'/exp

#16 'lixisenatide'/exp

#17 'dulaglutide'/exp

#18 'semaglutide'/exp

#19 'byetta'

#20 'bydureon'

#21 'victoza'

#22 'lyxumia'

#23 'adlyxin'

#24 'tanzeum'

#25 'eperzan'

#26 'trulicity'

#27 'ave 0010'

#28 'ozempic'

#29 'zp10a peptide'

#30 'zp10a peptide 1'

#31 'glp1 ra'

#32 #1 OR #2 OR #3 OR #4 OR #5 OR #6 OR #7 OR #8 OR #9 OR #10 OR #11 OR #12 OR #13 OR #14 OR #15 OR #16 OR #17 OR #18 OR #19 OR #20 OR #21 OR #22 OR #23 OR #24 OR #25 OR #26 OR #27 OR #28 OR #29 OR #30 OR #31

#33 'dipeptidyl peptidase iv inhibitor'

#34 'dipeptidyl peptidase iv inhibitors'

#35 'dipeptidyl peptidase iv inhibitor'/exp

#36 'dpp 4 inhibitor*'

#37 'dpp iv inhibitor*'

#38 'dpp4i'

#39 'dpp4 i'

#40 'dpp4 inhibitor*'

#41 'dppiv inhibitor*'

#42 'alogliptin'/exp

#43 'sitagliptin'/exp

#44 'gemigliptin'/exp

#45 'linagliptin'/exp

#46 'saxagliptin'/exp

#47 'vildagliptin'/exp

#48 'dutogliptin'/exp

#49 'teneligliptin'/exp

#50 'anagliptin'/exp

#51 'trelagliptin'/exp

#52 'pf-734200'/exp

#53 'melogliptin'/exp

#54 'evogliptin'/exp

#55 'retagliptin'

#56 'carmegliptin'/exp

#57 'lc15 0444'

#58 'tenelia'

#59 'da-1229'

#60 'omarigliptin'/exp

#61 'beskoa'

#62 'gemiglo'

#63 'trajenta'

#64 'kazano'

#65 'oseni'

#66 'nesina'

#67 'kombiglyze xr'

#68 'onglyza'

#69 'eucreas'

#70 'galvus'

#71 'juvisync'

#72 'janumet'

#73 'januvia'

#74 'liptin'

#75 'qtern'

#76 'vipidia'

#77 'vipdoment'

#78 'diabeglipt'

#79 'elant'

#80 'glucal'

#81 'teneglucon'

#82 'veriglip'

#83 'glipten'

#84 'suganon'

#85 'gliptin'/exp

#86 #33 OR #34 OR #35 OR #36 OR #37 OR #38 OR #39 OR #40 OR #41 OR #42 OR #43 OR #44 OR #45 OR #46 OR #47 OR #48 OR #49 OR #50 OR #51 OR #52 OR #53 OR #54 OR #55 OR #56 OR #57 OR #58 OR #59 OR #60 OR #61 OR #62 OR #63 OR #64 OR #65 OR #66 OR #67 OR #68 OR #69 OR #70 OR #71 OR #72 OR #73 OR #74 OR #75 OR #76 OR #77 OR #78 OR #79 OR #80 OR #81 OR #82 OR #83 OR #84 OR #85

#87 'sodium-glucose cotransporter 2'/exp

#88 'sodium-glucose cotransporter'/exp

#89 'Sodium-Glucose Transporter 2 inhibit*'

#90 'Sodium-Glucose cotransporter 2 Inhibit*'

#91 'Sodium-dependent glucose transporter 2 inhibit*'

#92 'Sodium glucose transporter 2 inhibit*'

#93 'SGLT2 inhibit*'

#94 'SGLT 2 inhibit*'

#95 'SGLTⅡ inhibit*'

#96 'SGLT Ⅱ inhibit*'

#97 'SGLT2i'.

#98 'Empagliflozin'/exp

#99 'Invokana'

#100 'Canagliflozin'/exp

#101 'Forxiga'

#102 'Dapagliflozin'/exp

#103 'Rongliflozin'/exp

#104 'Ipragliflozin'/exp

#105 'Luseogliflozin'/exp

#106 'Tofogliflozin'/exp

#107 'Ertugliflozin'/exp

#108 'Glyxambi'

#109 'Jardiance'

#110 'Bexagliflozin'/exp

#111 'Sotagliflozin'/exp

#112 'Licogliflozin'/exp

#113 'Sergliflozin'/exp

#114 'Henagliflozin'/exp

#115 'Remogliflozin'/exp

#116 'JNJ28431754'

#117 'TA7284'

#118 'BI10773'

#119 'SAR439954'

#120 'LX4211'

#121 'EGT0001442'

#122 'ASP1941'

#123 #87 OR #88 OR #89 OR #90 OR #91 OR #92 OR #93 OR #94 OR #95 OR #96 OR #97 OR #98 OR #99 OR #100 OR #101 OR #102 OR #103 OR #104 OR #105 OR #106 OR #107 OR #108 OR #109 OR #110 OR #111 OR #112 OR #113 OR #114 OR #115 OR #116 OR #117 OR #118 OR #119 OR #120 OR #121 OR #122

#124 'clinical trial'/exp OR 'controlled clinical trial'/exp OR 'randomized controlled trial'/exp

#125 random*

#126 'placebo'/exp

#127 'control'/exp

#128 'comparison group'

#129 'control group'/exp

#130 'registries'/exp

#131 'registration'/exp

#132 #124 OR #125 OR #126 OR #127 OR #128 OR #129 OR #130 OR #131

#133 #32 AND #132

#134 #32 AND #132 AND [humans]/lim

#135 #32 AND #132 AND [animals]/lim

#136 #135 NOT #134

#137 #133 NOT #136

#138 #86 AND #132

#139 #86 AND #132 AND [humans]/lim

#140 #86 AND #132 AND [animals]/lim

#141 #140 NOT #139

#142 #138 NOT #141

#143 #123 AND #132

#144 #123 AND #132 AND [humans]/lim

#145 #123 AND #132 AND [animals]/lim

#146 #145 NOT #144

#147 #143 NOT #146

#148 #137 AND ([article]/lim OR [article in press]/lim OR [conference abstract]/lim OR [conference paper]/lim)

#149 #142 AND ([article]/lim OR [article in press]/lim OR [conference abstract]/lim OR [conference paper]/lim)

#150 #147 AND ([article]/lim OR [article in press]/lim OR [conference abstract]/lim OR [conference paper]/lim)

#151 #148 AND [embase]/lim

#152 #149 AND [embase]/lim

#153 #150 AND [embase]/lim

#154 #151 OR #152 OR #153

**3. Cochrane Library：**

#1 (ZE "glucagon-like peptide 1") OR (ZE "glucagon-like peptide 1 administration & dosage") OR (ZE "glucagon-like peptide 1 agonists") OR (ZE "glucagon-like peptide 1 analogs & derivatives") OR (ZE "glucagon-like peptide 1 drug effects") OR (ZE "glucagon-like peptide 1 therapeutic use") OR (ZE "glucagon-like peptide-1 receptor agonists") OR (ZE "glucagon-like peptide 1 pharmacokinetics") OR (ZE "glucagon-like peptide 1 pharmacology")

#2 TX glucagon-like peptide 1

#3 TX Glucagon-Like Peptides 1

#4 TX glp-1 OR TX glp1

#5 TX exenatide

#6 TX liraglutide

#7 TX albiglutide

#8 TX taspoglutide

#9 TX lixisenatide

#10 TX dulaglutide

#11 TX semaglutide

#12 TX ZP10A peptide*

#13 TX GLP 1 Receptor Agonist*

#14 TX GLP 1 RA*

#15 TX GLP 1RA*

#16 TX Byetta OR TX Bydureon OR TX Victoza OR TX Lyxumia OR TX Adlyxin OR TX Tanzeum OR TX Eperzan OR TX Trulicity OR TX AVE 0010 OR TX Ozempic

#17 #1 OR #2 OR #3 OR #4 OR #5 OR #6 OR #7 OR #8 OR #9 OR #10 OR #11 OR #12 OR #13 OR #14 OR #15 OR #16

#18 (ZE "dipeptidyl-peptidase iv inhibitors") OR (ZE "dipeptidyl-peptidase iv inhibitors administration & dosage") OR (ZE "dipeptidyl-peptidase iv inhibitors adverse effects") OR (ZE "dipeptidyl-peptidase iv inhibitors pharmacokinetics") OR (ZE "dipeptidyl-peptidase iv inhibitors pharmacology") OR (ZE "dipeptidyl-peptidase iv inhibitors therapeutic use") OR (ZE "dipeptidyl-peptidase iv inhibitors toxicity")

#19 TX dipeptidyl-peptidase iv inhibitors OR TX dipeptidyl-peptidase iv inhibitor

#20 TX dipeptidyl-peptidase 4 inhibitor OR TX dipeptidyl-peptidase 4 inhibitors

#21 TX DPP 4 inhibit* OR TX DPP4 inhibit*

#22 TX DPP4i

#23 TX DPP IV inhibit* OR TX DPPIV inhibit*

#24 TX alogliptin

#25 TX sitagliptin

#26 TX vildagliptin

#27 TX saxagliptin

#28 TX linagliptin

#29 TX dutogliptin

#30 TX teneligliptin

#31 TX LC15 0444

#32 TX gemigliptin

#33 TX anagliptin

#34 TX Trelagliptin

#35 TX PF-734200

#36 TX Retagliptin

#37 TX Melogliptin

#38 TX Evogliptin

#39 TX Carmegliptin

#40 TX Januvia OR TX Janumet OR TX Juvisync OR TX Galvus OR TX Eucreas OR TX Onglyza OR TX Kombiglyze OR TX Qtern OR TX Nesina OR TX Oseni OR TX Kazano OR TX Vipidia OR TX Vipdoment OR TX Trajenta OR TX Gemiglo OR TX Beskoa OR TX Tenelia OR TX Diabeglipt OR TX Elant OR TX Glucal OR TX Teneglucon OR TX Veriglip OR TX Glipten OR TX Suganon

#41 #18 OR #19 OR #20 OR #21 OR #22 OR #23 OR #24 OR #25 OR #26 OR #27 OR #28 OR #29 OR #30 OR #31 OR #32 OR #33 OR #34 OR #35 OR #36 OR #37 OR #38 OR #39 OR #40

#42 (ZE "sodium-glucose cotransporter 2") OR (ZE " sodium-glucose cotransporter 2 administration & dosage") OR (ZE " sodium-glucose cotransporter 2 inhibitor") OR (ZE "sodium-glucose cotransporter 2 analogs & derivatives") OR (ZE " sodium-glucose cotransporter 2 drug effects") OR (ZE "sodium-glucose cotransporter 2 therapeutic use") OR (ZE " sodium-glucose cotransporter 2 pharmacokinetics") OR (ZE " sodium-glucose cotransporter 2 pharmacology")

#43 TX sodium-glucose transporter 2 OR TX sodium-dependent glucose transporter 2

#44 TX SGLT2 OR TX SGLT 2

#45 TX Empagliflozin

#46 TX Invokana

#47 TX Canagliflozin

#48 TX Forxiga

#49 TX Dapagliflozin

#50 TX Rongliflozin

#51 TX Ipragliflozin

#52 TX Luseogliflozin

#53 TX Tofogliflozin

#54 TX Ertugliflozin

#55 TX Glyxambi

#56 TX Jardiance

#57 TX Bexagliflozin

#58 TX Sotagliflozin

#59 TX Licogliflozin

#60 TX Sergliflozin

#61 TX Henagliflozin

#62 TX Remogliflozin

#63 TX JNJ28431754

#64 TX TA7284

#65 TX BI10773

#66 TX SAR439954

#67 TX LX4211

#68 TX EGT0001442

#69 TX ASP1941

#70 #42 OR #43 OR #44 OR #45 OR #46 OR #47 OR #48 OR #49 OR #50 OR #51 OR #52 OR #53 OR #54 OR #55 OR #56 OR #57 OR #58 OR #59 OR #60 OR #61 OR #62 OR #63 OR #64 OR #65 OR #66 OR #67 OR #68 OR #69

#71 #17 OR #41 OR #70

**4. Clinical Trials：**

exenatide OR liraglutide OR albiglutide OR taspoglutide OR lixisenatide OR dulaglutide OR semaglutide OR Byetta OR Bydureon OR Victoza OR Lyxumia OR Adlyxin OR Tanzeum OR Eperzan OR Trulicity OR ‘ZP10A peptide*’ OR ‘AVE 0010’ OR Ozempic OR sitagliptin OR Januvia OR Janumet OR Juvisync OR vildagliptin OR Galvus OR Eucreas OR ‘Galvus Met’ OR saxagliptin OR Onglyza OR ‘Kombiglyze XR’ OR Qtern OR alogliptin OR Nesina OR Oseni OR Kazano OR Vipidia OR Vipdoment OR linagliptin OR Trajenta OR gemigliptin OR Gemiglo OR anagliptin OR Beskoa OR teneligliptin OR Tenelia OR Diabeglipt OR Elant OR Glucal OR Teneglucon OR Veriglip OR Glipten OR Trelagliptin OR ‘PF 734200’ OR retagliptin OR Melogliptin OR evogliptin OR Suganon OR Carmegliptin OR "LC15 0444" OR ‘DA-1229’ OR omarigliptin OR gliptin* OR dutogliptin OR Empagliflozin OR Invokana OR Canagliflozin OR Forxiga OR Dapagliflozin OR Rongliflozin OR Ipragliflozin OR Luseogliflozin OR Tofogliflozin OR Ertugliflozin OR ‘Glyxambi’ OR Jardiance OR Bexagliflozin OR Sotagliflozin OR Licogliflozin OR Sergliflozin OR Henagliflozin OR Remogliflozin OR JNJ28431754 OR TA7284 OR BI10773 OR SAR439954 OR LX4211 OR EGT0001442 OR ASP1941 OR gliflozin*
