# Supplementary material for: Risk of Fracture With Dipeptidyl Peptidase-4 Inhibitors, Glucagon-like Peptide-1 Receptor Agonists, or Sodium-Glucose Cotransporter-2 Inhibitors in Patients With Type 2 Diabetes Mellitus: A Systematic Review and Network Meta-analysis Combining 177 Randomized Controlled Trials With a Median Follow-Up of 26 weeks
Source: Front Pharmacol. 2022 Jul 1;13:825417. doi: 10.3389/fphar.2022.825417 (PMC9285982; doi:10.3389/fphar.2022.825417)
Supplement: Supplementary file 4 [file DataSheet7.doc]

Supplementary appendix 8 Funnel plots based on fractures in different parts of the body


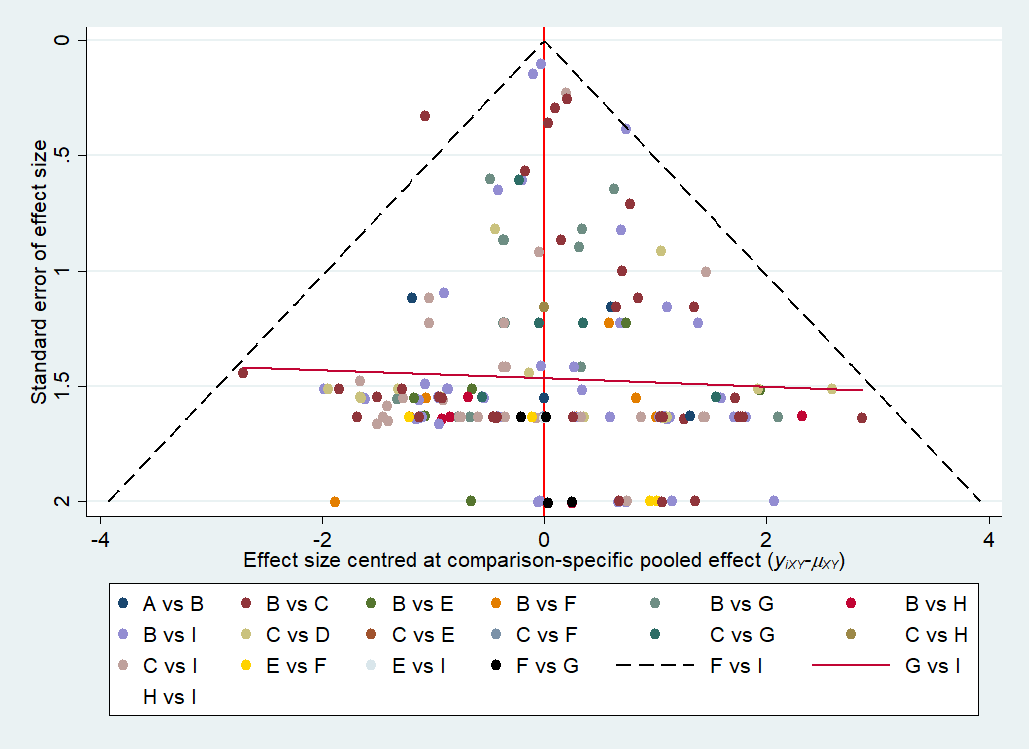


*( A : AGI; B: DPP-4i; C: GLP-1 RAs; D: Insulin; E: Metformin; F: SGLT-2i; G: Sulfonylurea; H: TZD; I: placebo)

Figure 1 Funnel plot for total fracture


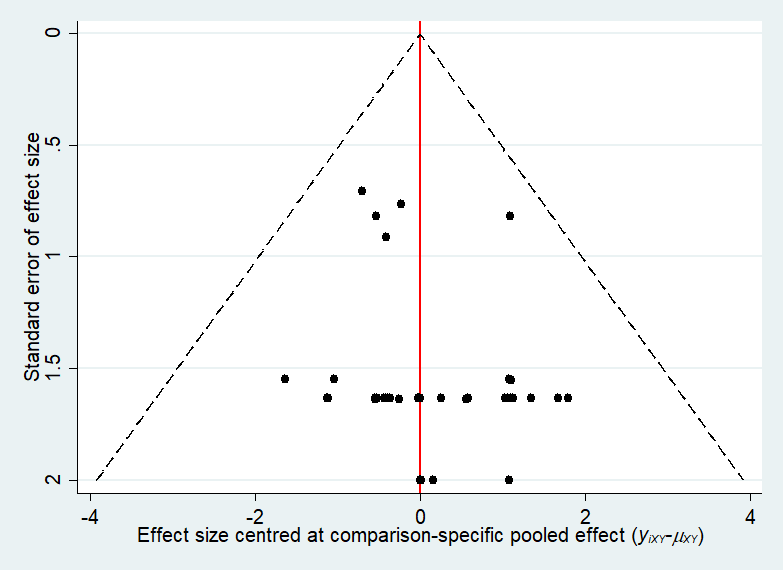


Figure 2 Funnel plot for spinal fracture


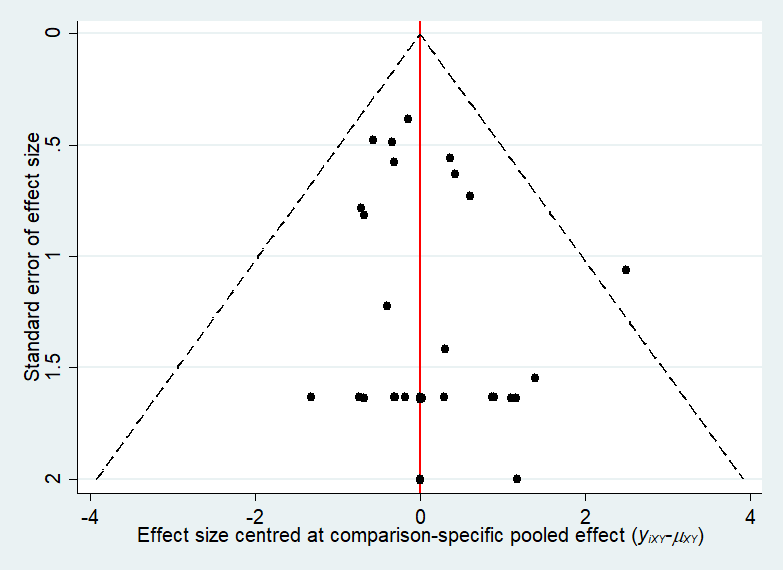


Figure 3 Funnel plot for hip fracture


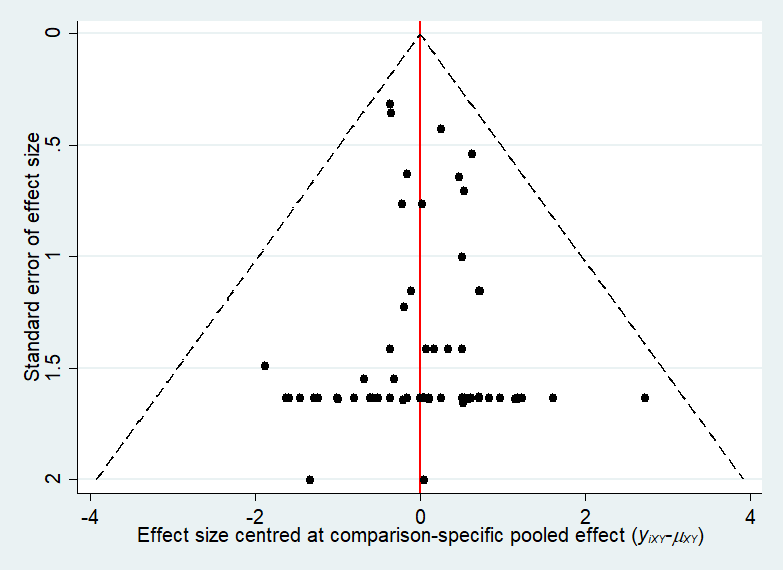


Figure 4 Funnel plot for upper limb fracture


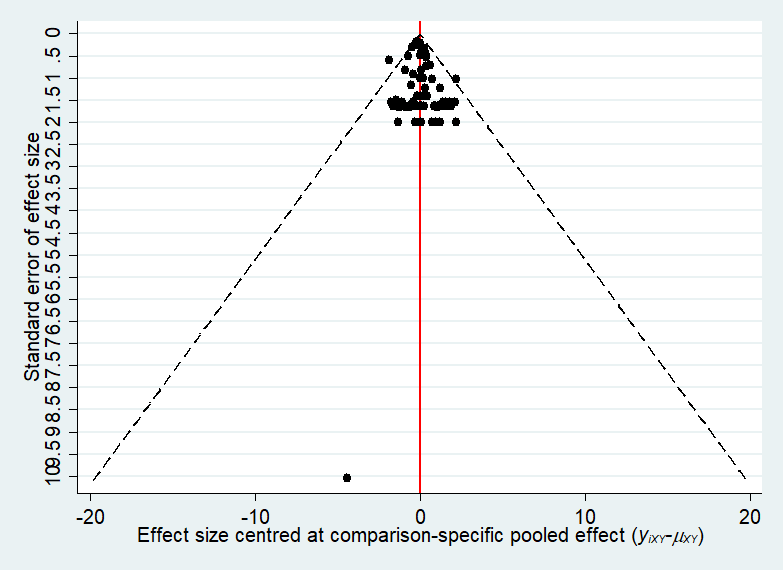


Figure 5 Funnel plot for lower limb fracture


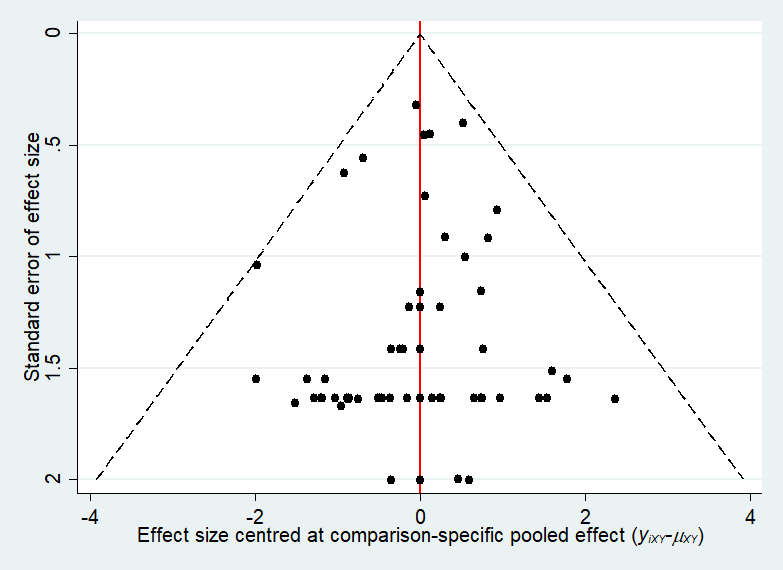


Figure 6 Funnel plot for other fracture
