# Supplementary material for: Risk of Fracture With Dipeptidyl Peptidase-4 Inhibitors, Glucagon-like Peptide-1 Receptor Agonists, or Sodium-Glucose Cotransporter-2 Inhibitors in Patients With Type 2 Diabetes Mellitus: A Systematic Review and Network Meta-analysis Combining 177 Randomized Controlled Trials With a Median Follow-Up of 26 weeks
Source: Front Pharmacol. 2022 Jul 1;13:825417. doi: 10.3389/fphar.2022.825417 (PMC9285982; doi:10.3389/fphar.2022.825417)
Supplement: Supplementary file 5 [file DataSheet4.doc]

Supplementary appendix 4 Risk of bias assessment

In the network meta-analysis of total fracture, the risk of bias of the included 177 studies was assessed by Cochrane Collaboration’s tool for assessing risk of bias. The result wa as follows.


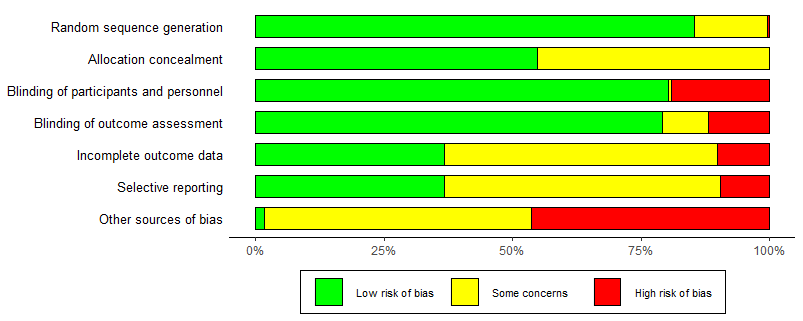


For the total 177 studies included in this analysis, majority of studies were assessed as “low risk” in random sequence generation (151/177, 85.3%), blinding of participants and personnel (142/177, 80.2%), and blinding of outcome assessment (140/177, 79.1%). Some studies were assessed as “low risk” in allocation concealment (97/177, 54.8%), complete outcome data (65/177, 36.7%), and selective reporting (65/177, 36.7%), only a few studies were rated as "high risk" in the above items. 46.3% of the studies were funded by enterprises. Overall, the risk of bias across evidence network was relatively low.
