# Supplementary material for: Risk of Fracture With Dipeptidyl Peptidase-4 Inhibitors, Glucagon-like Peptide-1 Receptor Agonists, or Sodium-Glucose Cotransporter-2 Inhibitors in Patients With Type 2 Diabetes Mellitus: A Systematic Review and Network Meta-analysis Combining 177 Randomized Controlled Trials With a Median Follow-Up of 26 weeks
Source: Front Pharmacol. 2022 Jul 1;13:825417. doi: 10.3389/fphar.2022.825417 (PMC9285982; doi:10.3389/fphar.2022.825417)
Supplement: Supplementary file 6 [file DataSheet5.docx]

Supplementary appendix 6 Subgroup analysis of fracture risk based on the different body parts

| DPP-4i |  |  |  |  |  |  |  |
| --- | --- | --- | --- | --- | --- | --- | --- |
| 0.76 (0.29,1.95) | GLP-1 RAs |  |  |  |  |  |  |
| 0.70 (0.17,2.86) | 0.92 (0.23,3.75) | SGLT-2i |  |  |  |  |  |
| 0.77 (0.13,4.45) | 1.01 (0.23,4.46) | 1.10 (0.14,8.44) | Insulin |  |  |  |  |
| 0.83 (0.03,20.52) | 1.10 (0.04,31.15) | 1.19 (0.04,39.61) | 1.09 (0.03,42.12) | Metformin |  |  |  |
| 1.61 (0.27,9.64) | 2.14 (0.30,15.38) | 2.31 (0.24,21.90) | 2.11 (0.18,24.91) | 1.94 (0.05,76.32) | Sulfonylurea |  |  |
| 2.14 (0.09,53.43) | 2.83 (0.13,63.20) | 3.06 (0.10,89.62) | 2.79 (0.09,87.32) | 2.57 (0.03,241.79) | 1.32 (0.03,51.32) | TZD |  |
| 0.69 (0.32,1.49) | 0.91 (0.47,1.77) | 0.99 (0.29,3.41) | 0.90 (0.18,4.57) | 0.83 (0.03,22.45) | 0.43 (0.06,2.88) | 0.32 (0.01,7.47) | Placebo |

Figure 1 Odds ratio with 95%CI of subgroup analysis for spinal fracture

| DPP-4i |  |  |  |  |  |  |
| --- | --- | --- | --- | --- | --- | --- |
| 0.52 (0.24,1.12) | GLP-1 RAs |  |  |  |  |  |
| 0.70 (0.32,1.51) | 1.34 (0.57,3.17) | SGLT-2i |  |  |  |  |
| 0.75 (0.03,20.24) | 1.44 (0.06,35.45) | 1.07 (0.04,29.65) | Insulin |  |  |  |
| 0.14 (0.01,1.88) | 0.27 (0.02,3.70) | 0.20 (0.02,2.57) | 0.18 (0.00,11.69) | Metformin |  |  |
| 0.78 (0.16,3.83) | 1.50 (0.35,6.47) | 1.12 (0.21,5.86) | 1.04 (0.03,35.26) | 5.63 (0.28,111.70) | Sulfonylurea |  |
| 0.79 (0.49,1.28) | 1.52 (0.83,2.78) | 1.14 (0.61,2.10) | 1.06 (0.04,27.55) | 5.72 (0.44,74.07) | 1.02 (0.22,4.72) | Placebo |

Figure 2 Odds ratio with 95%CI of subgroup analysis for hip fracture

| DPP-4i |  |  |  |  |  |  |
| --- | --- | --- | --- | --- | --- | --- |
| 1.21 (0.72,2.05) | GLP-1 RAs |  |  |  |  |  |
| 1.84 (0.79,4.29) | 1.52 (0.63,3.64) | SGLT-2i |  |  |  |  |
| 0.71 (0.19,2.60) | 0.59 (0.18,1.92) | 0.39 (0.09,1.69) | Insulin |  |  |  |
| 1.17 (0.18,7.75) | 0.97 (0.15,6.41) | 0.64 (0.09,4.39) | 1.65 (0.18,15.39) | Metformin |  |  |
| 1.18 (0.53,2.62) | 0.97 (0.42,2.24) | 0.64 (0.23,1.77) | 1.65 (0.39,7.06) | 1.00 (0.13,7.44) | Sulfonylurea |  |
| 1.08 (0.73,1.59) | 0.89 (0.60,1.31) | 0.58 (0.26,1.30) | 1.51 (0.43,5.28) | 0.92 (0.14,6.02) | 0.91 (0.41,2.07) | Placebo |

Figure 3 Odds ratio with 95%CI of subgroup analysis for upper limb fracture

| DPP-4i |  |  |  |  |  |  |  |
| --- | --- | --- | --- | --- | --- | --- | --- |
| 1.23 (0.86,1.76) | GLP-1 RAs |  |  |  |  |  |  |
| 1.35 (0.88,2.07) | 1.10 (0.68,1.78) | SGLT-2i |  |  |  |  |  |
| 1.06 (0.44,2.54) | 0.86 (0.39,1.91) | 0.78 (0.31,2.00) | Insulin |  |  |  |  |
| 0.95 (0.15,6.06) | 0.77 (0.12,5.10) | 0.71 (0.11,4.72) | 0.90 (0.12,6.98) | Metformin |  |  |  |
| 1.34 (0.76,2.35) | 1.09 (0.60,1.99) | 0.99 (0.51,1.93) | 1.27 (0.47,3.44) | 1.41 (0.20,9.76) | Sulfonylurea |  |  |
| 0.73 (0.15,3.53) | 0.60 (0.12,2.91) | 0.54 (0.11,2.73) | 0.69 (0.12,4.10) | 0.77 (0.07,8.74) | 0.55 (0.10,2.88) | TZD |  |
| 1.11 (0.87,1.42) | 0.90 (0.66,1.24) | 0.82 (0.57,1.20) | 1.05 (0.44,2.48) | 1.17 (0.18,7.55) | 0.83 (0.46,1.48) | 1.51 (0.31,7.28) | Placebo |

Figure 4 Odds ratio with 95%CI of subgroup analysis for lower limb fracture

| DPP-4i |  |  |  |  |  |  |  |
| --- | --- | --- | --- | --- | --- | --- | --- |
| 1.09 (0.61,1.95) | GLP-1 RAs |  |  |  |  |  |  |
| 0.85 (0.45,1.60) | 0.78 (0.41,1.49) | SGLT-2i |  |  |  |  |  |
| 0.94 (0.27,3.28) | 0.87 (0.29,2.61) | 1.11 (0.31,4.01) | Insulin |  |  |  |  |
| 0.82 (0.07,9.95) | 0.75 (0.06,9.54) | 0.97 (0.08,11.96) | 0.87 (0.05,13.86) | Metformin |  |  |  |
| 0.94 (0.27,3.31) | 0.86 (0.25,3.03) | 1.11 (0.31,3.99) | 1.00 (0.19,5.30) | 1.15 (0.07,18.30) | Sulfonylurea |  |  |
| 1.76 (0.29,10.80) | 1.62 (0.26,9.97) | 2.08 (0.32,13.41) | 1.87 (0.22,15.67) | 2.15 (0.10,46.30) | 1.87 (0.21,16.31) | TZD |  |
| 0.71 (0.46,1.09) | 0.65 (0.42,1.01) | 0.84 (0.51,1.39) | 0.76 (0.23,2.48) | 0.87 (0.07,10.65) | 0.76 (0.22,2.62) | 0.40 (0.07,2.44) | Placebo |

Figure 5 Odds ratio with 95%CI of subgroup analysis for other fractures

Note: Results of direct comparisons were listed in the upper triangle, and the estimation was calculated as the row-defining treatment compared with the column-defining treatment. Results of network meta-analysis were listed in the lower triangle, and the estimation was calculated as the column-defining treatment compared with the row-defining treatment.
