# Supplementary material for: Risk of Fracture With Dipeptidyl Peptidase-4 Inhibitors, Glucagon-like Peptide-1 Receptor Agonists, or Sodium-Glucose Cotransporter-2 Inhibitors in Patients With Type 2 Diabetes Mellitus: A Systematic Review and Network Meta-analysis Combining 177 Randomized Controlled Trials With a Median Follow-Up of 26 weeks
Source: Front Pharmacol. 2022 Jul 1;13:825417. doi: 10.3389/fphar.2022.825417 (PMC9285982; doi:10.3389/fphar.2022.825417)
Supplement: Supplementary file 7 [file DataSheet8.doc]

Supplementary appendix 9 Evidence level assessment by GRADE

The Grading of Recommendations, Assessment, Development and Evaluation (GRADE) system was used to assess the quality of the evidence. The CINeMA (https://cinema.ispm.unibe.ch/) was used to complete the whole process.


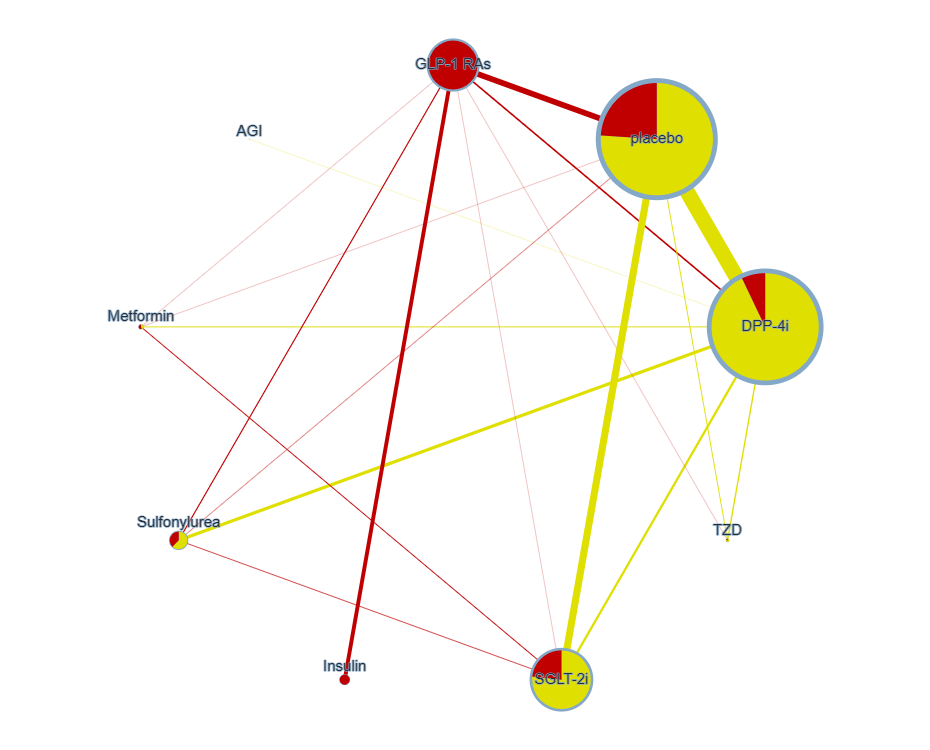


Figure 1 CINeMA netplot with risk of bias


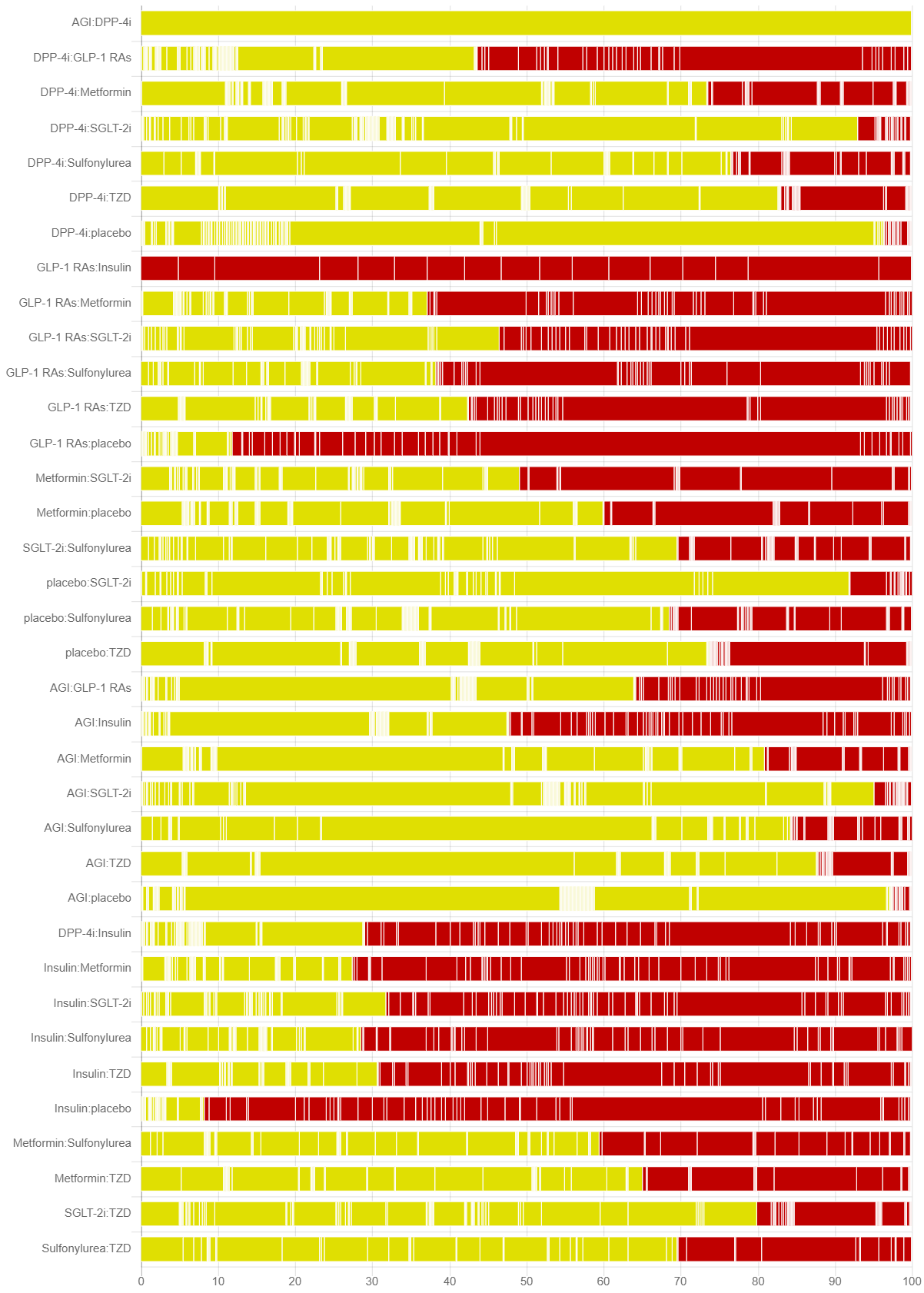


Figure 2 CINeMA risk of bias chart

Table 1 CINEMA report

| Comparison | Number of studies | Within-study bias | Reporting bias | Indirectness | Imprecision | Heterogeneity | Incoherence | Confidence rating |
| --- | --- | --- | --- | --- | --- | --- | --- | --- |
| Mixed evidence | | | | | | | | |
| AGI:DPP-4i | 1 | Some concerns | Undetected | No concerns | Major concerns | No concerns | No concerns | Moderate |
| DPP-4i:GLP-1 RAs | 7 | Major concerns | Undetected | No concerns | Major concerns | No concerns | No concerns | Moderate |
| DPP-4i:Metformin | 4 | Some concerns | Undetected | No concerns | Major concerns | No concerns | No concerns | Moderate |
| DPP-4i:SGLT-2i | 10 | Some concerns | Undetected | No concerns | Major concerns | No concerns | No concerns | Moderate |
| DPP-4i:Sulfonylurea | 14 | Some concerns | Undetected | No concerns | Major concerns | No concerns | No concerns | Moderate |
| DPP-4i:TZD | 6 | Some concerns | Undetected | No concerns | Major concerns | No concerns | No concerns | Moderate |
| DPP-4i:placebo | 67 | Some concerns | Undetected | No concerns | Major concerns | No concerns | No concerns | Moderate |
| GLP-1 RAs:Insulin | 17 | Major concerns | Undetected | No concerns | Major concerns | No concerns | No concerns | Moderate |
| GLP-1 RAs:Metformin | 1 | Major concerns | Undetected | No concerns | Major concerns | No concerns | No concerns | Moderate |
| GLP-1 RAs:SGLT-2i | 1 | Major concerns | Undetected | No concerns | Major concerns | No concerns | No concerns | Moderate |
| GLP-1 RAs:Sulfonylurea | 5 | Major concerns | Undetected | No concerns | Major concerns | No concerns | No concerns | Moderate |
| GLP-1 RAs:TZD | 1 | Major concerns | Undetected | No concerns | Major concerns | No concerns | No concerns | Moderate |
| GLP-1 RAs:placebo | 25 | Major concerns | Undetected | No concerns | Major concerns | No concerns | No concerns | Moderate |
| Metformin:SGLT-2i | 4 | Major concerns | Undetected | No concerns | Major concerns | No concerns | No concerns | Moderate |
| Metformin:placebo | 1 | Some concerns | Undetected | No concerns | Major concerns | No concerns | No concerns | Moderate |
| SGLT-2i:Sulfonylurea | 3 | Some concerns | Undetected | No concerns | Major concerns | No concerns | No concerns | Moderate |
| placebo:SGLT-2i | 31 | Some concerns | Undetected | No concerns | Major concerns | No concerns | No concerns | Moderate |
| placebo:Sulfonylurea | 2 | Some concerns | Undetected | No concerns | Major concerns | No concerns | No concerns | Moderate |
| placebo:TZD | 4 | Some concerns | Undetected | No concerns | Major concerns | No concerns | No concerns | Moderate |
| Indirect evidence | | | | | | | | |
| AGI:GLP-1 RAs | 0 | Some concerns | Undetected | No concerns | Major concerns | No concerns | No concerns | Moderate |
| AGI:Insulin | 0 | Major concerns | Undetected | No concerns | Major concerns | No concerns | No concerns | Moderate |
| AGI:Metformin | 0 | Some concerns | Undetected | No concerns | Major concerns | No concerns | No concerns | Moderate |
| AGI:SGLT-2i | 0 | Some concerns | Undetected | No concerns | Major concerns | No concerns | No concerns | Moderate |
| AGI:Sulfonylurea | 0 | Some concerns | Undetected | No concerns | Major concerns | No concerns | No concerns | Moderate |
| AGI:TZD | 0 | Some concerns | Undetected | No concerns | Major concerns | No concerns | No concerns | Moderate |
| AGI:placebo | 0 | Some concerns | Undetected | No concerns | Major concerns | No concerns | No concerns | Moderate |
| DPP-4i:Insulin | 0 | Major concerns | Undetected | No concerns | Major concerns | No concerns | No concerns | Moderate |
| Insulin:Metformin | 0 | Major concerns | Undetected | No concerns | Major concerns | No concerns | No concerns | Moderate |
| Insulin:SGLT-2i | 0 | Major concerns | Undetected | No concerns | Major concerns | No concerns | No concerns | Moderate |
| Insulin:Sulfonylurea | 0 | Major concerns | Undetected | No concerns | Major concerns | No concerns | No concerns | Moderate |
| Insulin:TZD | 0 | Major concerns | Undetected | No concerns | Major concerns | No concerns | No concerns | Moderate |
| Insulin:placebo | 0 | Major concerns | Undetected | No concerns | Major concerns | No concerns | No concerns | Moderate |
| Metformin:Sulfonylurea | 0 | Some concerns | Undetected | No concerns | Major concerns | No concerns | No concerns | Moderate |
| Metformin:TZD | 0 | Some concerns | Undetected | No concerns | Major concerns | No concerns | No concerns | Moderate |
| SGLT-2i:TZD | 0 | Some concerns | Undetected | No concerns | Major concerns | No concerns | No concerns | Moderate |
| Sulfonylurea:TZD | 0 | Some concerns | Undetected | No concerns | Major concerns | No concerns | No concerns | Moderate |

As shown in Table 1, the quality of most studies was moderate. The quality of evidence in total fracture was rated as moderate
