# Supplementary material for: Risk of Fracture With Dipeptidyl Peptidase-4 Inhibitors, Glucagon-like Peptide-1 Receptor Agonists, or Sodium-Glucose Cotransporter-2 Inhibitors in Patients With Type 2 Diabetes Mellitus: A Systematic Review and Network Meta-analysis Combining 177 Randomized Controlled Trials With a Median Follow-Up of 26 weeks
Source: Front Pharmacol. 2022 Jul 1;13:825417. doi: 10.3389/fphar.2022.825417 (PMC9285982; doi:10.3389/fphar.2022.825417)
Supplement: Supplementary file 8 [file DataSheet2.docx]

Supplementary appendix 2 Characteristics of the 177 studies included in the network meta-analysis

| **No.** | **Study ID** | **Duration**  **(week)** | **Treatment** | **Control** | **Background medication** | **Sample size** | **Mean age**  **(year)** | **Mean HbA1c**  **(%)** | **Mean duration of T2DM**  **(year)** |
| --- | --- | --- | --- | --- | --- | --- | --- | --- | --- |
| 1 | Ahmann A 2015 | 26w | GLP-1 RAs | placebo | - | 450 | 59.30 | - | 12.10 |
| 2 | Alba M 2013 | 12w | DPP-4i | TZD; placebo | - | 159 | 53.77 | 7.90 | 2.40 |
| 3 | Allegretti A S 2019 | 24w | SGLT-2i | placebo | - | 312 | 69.60 | 7.98 | 15.91 |
| 4 | Arakaki RF 2014 | 24w | GLP-1 RAs | insulin | - | 510 | - | - | - |
| 5 | Araki E 2016 | 16w | SGLT-2i | placebo | insulin or DPP-4i | 182 | 58.00 | 8.34 | 14.97 |
| 6 | Arechavaleta R 2009 | 30w | DPP-4i | sulfonylurea | metformin | 1035 | 56.30 | 7.50 | - |
| 7 | Arjona Ferreira JC 2013 54w | 54w | DPP-4i | sulfonylurea | - | 423 | 64.20 | 7.78 | - |
| 8 | Arjona Ferreira JC 2013 54w-pc | 54w | DPP-4i | sulfonylurea | - | 129 | 59.50 | 7.80 | 17.50 |
| 9 | Aschner P 2010 | 24w | DPP-4i | metformin | - | 1050 | 56.00 | 7.30 | 2.35 |
| 10 | AWARD-1 Wysham C 2014 | 26w | GLP-1 RAs | placebo | metformin+  pioglitazone | 976 | 55.65 | - | 8.76 |
| 11 | AWARD-2 Giorgino F 2015 | 78w | GLP-1 RAs | insulin | metformin; glimepiride | 810 | 56.66 | - | 9.10 |
| 12 | AWARD-3 Umpierrez G 2014 | 52w | GLP-1 RAs | metformin | diet+exercise | 807 | 55.56 | - | 2.63 |
| 13 | AWARD-4 Blonde L 2015 | 52w | GLP-1 RAs | insulin | insulin | 884 | 59.36 | - | 12.73 |
| 14 | AWARD-5 Nauck M 2014 | 104w | GLP-1 RAs | DPP-4i | diet+exercise  /monotherapy /metformin+  monotherapy | 921 | 53.91 | - | 7.15 |
| 15 | AWARD-5 Weinstock RS | 104w | GLP-1 RAs | DPP-4i | metformin | 921 | 54.00 | 8.10 | 7.00 |
| 16 | AWARD-7 2017 | 26w | GLP-1 RAs | insulin | insulin | 576 | 64.60 | - | 18.08 |
| 17 | AWARD-8 Dungan 2016 | 24w | GLP-1 RAs | placebo | - | 299 | 57.83 | - | 7.60 |
| 18 | AWARD-9 Pozzilli P 2017 | 28w | GLP-1 RAs | placebo | insulin | 300 | 60.40 | - | 13.15 |
| 19 | Balis D. A 2014 | 52w | SGLT-2i | placebo | - | 584 | 55.40 | - | - |
| 20 | Barnett AH 2013 24w | 24w | DPP-4i | placebo | oral antidiabetic  drugs | 241 | 74.90 | 7.78 | - |
| 21 | Barnett AH 2013 24w-1 | 24w | DPP-4i | placebo | insulin/insulin+  metformin | 455 | 57.20 | 8.70 | 12.00 |
| 22 | Barnett AH 2013 52w | 52w | DPP-4i | placebo | insulin/insulin+  metformin | 455 | 57.20 | 8.70 | 12.00 |
| 23 | Barnett AH 2014 | 52w | SGLT-2i | placebo | - | 738 | 63.90 | - | - |
| 24 | Barzilai N 2011 | 24w | DPP-4i | placebo | OAD | 206 | 71.90 | 7.80 | 7.10 |
| 25 | Bode B 2015 | 104w | SGLT-2i | placebo | - | 714 | 63.60 | 7.70 | 11.70 |
| 26 | Bosi E 2009 | 24w | DPP-4i | placebo | metformin | 879 | 52.57 | 8.63 | 2.00 |
| 27 | Bosi E 2011 | 52w | DPP-4i | placebo | metformin+  pioglitazone | 803 | 55.10 | 8.20 | 7.16 |
| 28 | Buse JB 2011 | 30w | GLP-1 RAs | placebo | Insulin glargine +/-metformin/TZD | 259 | 59.01 | - | 12.00 |
| 29 | CANTATA-D Trial Lavalle-Gonzalez FJ 2013 | 52w | DPP-4i;  SGLT-2i | placebo | metformin | 1284 | 55.40 | 7.90 | 6.90 |
| 30 | CANTATA-D2 Schernthaner G 2013 | 52w | DPP-4i | SGLT-2i | metformin+ sulfonylurea | 755 | 56.70 | 8.10 | 9.60 |
| 31 | CANTATA-MP Trial Forst T 2014 | 52w | DPP-4i | SGLT-2i | metformin+ pioglitazone | 342 | 57.40 | 7.90 | 10.50 |
| 32 | Cefalu WT 2013 | 52w | SGLT-2i | sulfonylurea | - | 1450 | 56.20 | - | - |
| 33 | Cefalu WT 2015 | 24w | SGLT-2i | placebo | - | 914 | 62.90 | 8.13 | - |
| 34 | Charbonnel B 2006 | 104w | DPP-4i | Sulfonylurea | metformin+TZD | 701 | 54.50 | 8.00 | 6.20 |
| 35 | Charbonnel B 2013 | 26w | GLP-1 RAs | DPP-4i | metformin | 653 | 57.30 | - | - |
| 36 | Dagogo-Jack S 2017 | 52w | SGLT-2i | placebo | metformin; sitagliptin | 462 | 59.10 | 8.03 | - |
| 37 | Dandona P 2017 | 24w | SGLT-2i | placebo | insulin | 778 | 42.50 | 8.53 | 20.30 |
| 38 | DeFronzo RA 2012 | 26w | DPP-4i | TZD; placebo | metformin | 1554 | 54.50 | 8.55 | 6.24 |
| 39 | Diamant M 2014 | 30w | GLP-1 RAs | insulin | insluin/metformin | 627 | 59.50 | - | - |
| 40 | Dobs AS 2013 | 54w | DPP-4i | placebo | metformin | 262 | 54.50 | 8.80 | 9.30 |
| 41 | DUAL-I Holst JJ 2016 | 26w | GLP-1 RAs | insulin | metformin  pioglitazone | 1663 | 55.00 | - | - |
| 42 | ENDURE NCT00856284 2013 | 104w | DPP-4i | sulfonylurea | metformin | 2639 | 55.40 | 7.60 | 5.52 |
| 43 | EUREXA Gallwitz B 2012 | 156w | GLP-1 RAs | sulfonylurea | metformin | 1019 | 56.40 | - | 5.65 |
| 44 | EXAMINE White WB 2013 | 76w | DPP-4i | placebo | oral antidiabetic  drugs | 5380 | 61.00 | 8.00 | 7.20 |
| 45 | Ferrannini E 2013 | 12w | SGLT-2i | metformin;  placebo | - | 406 | 57.50 | 7.90 | - |
| 46 | Ferrannini E 2013 | 78w | DPP-4i  ;SGLT-2i | placebo | metformin | 444 | - | - | - |
| 47 | Fonseca V 2007 | 24w | DPP-4i | placebo | insulin | 296 | 59.20 | 8.40 | 14.70 |
| 48 | Fonseca V 2013 | 26w | DPP-4i | placebo | metformin+ pioglitazone | 313 | 56.10 | 8.70 | 9.80 |
| 49 | Frederich R 2012 | 24w | DPP-4i | placebo | - | 294 | 55.00 | 7.90 | 1.70 |
| 50 | Frias JP 2017 | 28w | GLP-1 RAs | SGLT-2i | - | 683 | 54.20 | - | - |
| 51 | Fulcher G 2015 | 416w | SGLT-2i | placebo | sulfonylurea | 4327 | 62.40 | - | - |
| 52 | Gallwitz B 2011 | 26w | GLP-1 RAs | insulin | metformin/  sulfonylurea | 480 | 57.10 | - | 5.00 |
| 53 | Gallwitz B 2012 | 104w | DPP-4i | sulfonylurea | metformin | 1551 | 59.80 | 7.70 | - |
| 54 | GENERATION Schernthaner G 2015 | 52w | DPP-4i | sulfonylurea | metformin | 720 | 72.60 | 7.60 | 7.60 |
| 55 | GetGoal-F1 Bolli G 2013 | 24w | GLP-1 RAs | placebo | metformin | 321 | 56.10 | - | 6.00 |
| 56 | GetGoal-L Riddle MC 2013 | 24w | GLP-1 RAs | placebo | metformin+/-sulfonylurea+/-TZD | 495 | 57.20 | - | 12.50 |
| 57 | GetGoal-L-Asia Seino Y 2012 | 24w | GLP-1 RAs | placebo | insulin/  sulfonylurea | 311 | 58.40 | - | 13.92 |
| 58 | GetGoal-M Ahren B 2013 | 24w | GLP-1 RAs | placebo | metformin | 380 | 54.70 | - | 6.11 |
| 59 | GetGoal-Mono Fonseca VA 2012 | 12w | GLP-1 RAs | placebo | - | 361 | 53.70 | - | 1.10 |
| 60 | GetGoal-O Meneilly GS 2017 | 24w | GLP-1 RAs | placebo | - | 350 | 74.20 | - | 14.10 |
| 61 | GetGoal-P Pinget M 2013 | 24w | GLP-1 RAs | placebo | pioglitazone+/-metformin | 484 | 55.80 | - | 8.10 |
| 62 | GetGoal-S Rosenstock J 2014 | 24w | GLP-1 RAs | placebo | metformin+/-sulfonylurea | 859 | 57.20 | - | 9.33 |
| 63 | Gill A 2010 | 12w | GLP-1 RAs | placebo | metformin/  metformin+TZD | 54 | 55.43 | - | 6.52 |
| 64 | Goke B 2013 | 104w | DPP-4i | sulfonylurea | metformin | 858 | 57.60 | 7.65 | 5.45 |
| 65 | Goldstein BJ 2007 | 104w | DPP-4i | placebo | metformin | 1091 | 53.50 | 8.80 | 4.50 |
| 66 | Grunberger G 2018 | 52w | SGLT-2i | placebo | - | 467 | 67.30 | 8.15 | - |
| 67 | Haak T 2013 | 54w | DPP-4i | placebo | metformin | 395 | 55.80 | 7.50 | - |
| 68 | Hadjadj S 2016 | 24w | SGLT-2i | metformin | - | 680 | 52.86 | 8.69 | - |
| 69 | Haering HU 2013 | 24w | SGLT-2i | placebo | metformin+  sulfonylurea | 666 | 57.10 | 8.10 | - |
| 70 | Haering HU 2015 | 76w | SGLT-2i | placebo | metformin+  sulfonylurea | 666 | 57.10 | 8.10 | - |
| 71 | Halvorsen Y 2019 | 24w | DPP-4i | SGLT-2i | metformin | 384 | 59.40 | 7.99 | 8.79 |
| 72 | HARMONY-2 Nauck MA 2016 | 156w | GLP-1 RAs | placebo | - | 301 | 52.91 | - | 3.97 |
| 73 | HARMONY-3 Ahren B 2014 | 156w | GLP-1 RAs;  DPP-4i | sulfonylurea; placebo | metformin | 1012 | 54.50 | - | 6.01 |
| 74 | HARMONY-4 Weissman PN 2014 | 156w | GLP-1 RAs | insulin | metformin/  sulfonylurea/  metformin+  sulfonylurea | 745 | 55.44 | - | 8.74 |
| 75 | HARMONY-5 Home PD 2015 | 156w | GLP-1 RAs | TZD; placebo | metformin+  glimepride | 663 | 55.20 | - | 8.90 |
| 76 | HARMONY-6 Rosenstock J 2014 | 60w | GLP-1 RAs | insulin | insulin | 566 | 55.60 | - | 11.00 |
| 77 | Heine RJ 2005 | 26w | GLP-1 RAs | insulin | metformin+  sulfonylurea | 549 | 58.91 | - | 9.56 |
| 78 | Henry RR 2012 24w NCT00643851 | 24w | SGLT-2i | metformin | - | 598 | 52.00 | 9.17 | 1.60 |
| 79 | Henry RR 2012 24w NCT00859898 | 24w | SGLT-2i | metformin | - | 638 | 51.60 | 9.06 | 2.07 |
| 80 | Hirose T 2015 | 12w | DPP-4i | placebo | - | 156 | 59.30 | 8.10 | 12.80 |
| 81 | Hollander P 2019 | 104w | SGLT-2i | sulfonylurea | - | 1315 | 58.20 | 7.80 | 7.40 |
| 82 | Hollander PL 2011 | 76w | DPP-4i | placebo | TZD | 565 | 54.00 | 8.30 | 5.20 |
| 83 | Inagaki N 2012 | 26w | GLP-1 RAs | insulin | Biguanides + TZD | 427 | 57.10 | - | 8.86 |
| 84 | INTERVAL Strain WD 2013 | 24w | DPP-4i | placebo | oral antidiabetic  drugs | 278 | 74.75 | 7.90 | 11.65 |
| 85 | Iwamoto Y 2010 | 12w | DPP-4i | AGI | - | 380 | 59.20 | 7.60 | 5.40 |
| 86 | Jabbour SA 2014 | 24w | SGLT-2i | placebo | sitagliptin; metformin | 451 | 54.90 | 7.93 | - |
| 87 | Jadzinsky M 2009 | 24w | DPP-4i | placebo | metformin | 1306 | 51.99 | 9.50 | 1.70 |
| 88 | Jaiswal M 2015 | 78w | GLP-1 RAs | Insulin | - | 46 | 53.00 | - | 7.48 |
| 89 | Jardine MJ 2017 | 240w | SGLT-2i | placebo | - | 4397 | 63.00 | - | - |
| 90 | Ji L 2014 | 24w | SGLT-2i | placebo | - | 393 | 51.30 | 8.26 | 1.38 |
| 91 | Kadowaki T 2013 | 12w | DPP-4i | TZD; placebo | - | 324 | 58.20 | 7.80 | 6.20 |
| 92 | Kadowaki T 2014 | 12w | SGLT-2i | placebo | - | 547 | 57.50 | - | - |
| 93 | Kaku K 2016 | 52w | GLP-1 RAs | placebo | oral antidiabetic  drugs | 360 | 59.50 | - | 8.02 |
| 94 | Kashiwagi A 2011 | 12w | DPP-4i | placebo | TZD(pioglitazone) | 134 | 58.40 | 8.10 | 7.90 |
| 95 | Kohan DE 2013 | 104w | SGLT-2i | placebo | insulin | 252 | 67.01 | 8.35 | 16.94 |
| 96 | LEAD-2 Nauck M 2009 | 104w | GLP-1 RAs | sulfonylurea; placebo | metformin | 1087 | 56.70 | - | 7.40 |
| 97 | LEAD-3 Garber A 2011 | 104w | GLP-1 RAs | sulfonylurea | metformin/  sulfonylurea/  biguanide/  metformin+TZD | 746 | 53.00 | - | 5.40 |
| 98 | LEADER Marre M 2015 | 262w | GLP-1 RAs | placebo | insulin | 9340 | 64.30 | - | - |
| 99 | Leiter LA 2014 | 52w | GLP-1 RAs | DPP-4 | oral antidiabetic  drugs | 495 | - | - | - |
| 100 | LixiLan-O Davies MJ 2017 | 30w | GLP-1 RAs | insulin | metformin | 1169 | 58.40 | - | 8.80 |
| 101 | MARLINA-T2D study Groop P 2015 | 24w | DPP-4i | placebo | - | 360 | 60.60 | - | - |
| 102 | Mathieu C 2014 | 26w | GLP-1 RAs | insulin | metformin+ IDegLira | 177 | 61.00 | - | 12.35 |
| 103 | Mathieu C 2015 | 24w | DPP-4i | placebo | insulin+metformin | 658 | 58.80 | - | 13.50 |
| 104 | McGill JB 2013 | 52w | DPP-4i | placebo | insulin | 133 | 64.40 | 8.20 | - |
| 105 | MEXELIN 2013 | 24w | GLP-1 RAs | placebo | metformin/insulin | 34 | - | - | - |
| 106 | Mita T 2016 104w | 104w | DPP-4i | placebo | - | 341 | 64.60 | 7.30 | 8.60 |
| 107 | Mita T 2016 104w2 | 104w | DPP-4i | placebo | - | 282 | 63.70 | 8.05 | 17.25 |
| 108 | Mu YM 24w 2016 | 24w | DPP-4i | metformin | - | 436 | 51.43 | - | - |
| 109 | Nauck M 2007 | 104w | DPP-4i | sulfonylurea | metformin | 1172 | 56.70 | 7.70 | 6.40 |
| 110 | Nauck MA 2007 52w | 52w | GLP-1 RAs | insulin | metformin/  sulfonylurea | 501 | 58.70 | - | 9.90 |
| 111 | Nauck MA 2007 52w-pc | 52w | DPP-4i | sulfonylurea | metformin | 1172 | 56.70 | 7.70 | 6.40 |
| 112 | NCT00121667 2014 | 206w | DPP-4i | placebo | metformin+ pioglitazone | 743 | 54.57 | 8.05 | - |
| 113 | NCT00295633 2014 | 24w | DPP-4i | placebo | metformin+TZD | 565 | 54.04 | 8.26 | - |
| 114 | NCT00374907 2008 | 116w | DPP-4i | placebo | metformin | 36 | 55.50 | - | - |
| 115 | NCT00601250 2009 | 24w | DPP-4i | placebo | metformin | 700 | 56.50 | 8.08 | - |
| 116 | NCT00602472 2014 | 24w | DPP-4i | placebo | metformin+ sulfonylurea | 1055 | 58.10 | 8.14 | - |
| 117 | NCT00661362 2012 | 24w | DPP-4i | placebo | metformin | 570 | 54.05 | 7.92 | - |
| 118 | NCT00722371 2010 | 54w | DPP-4i | TZD | metformin+TZD  (pioglitazone) | 1615 | - | - | - |
| 119 | NCT00798161 2010 24w-1 | 24w | DPP-4i | placebo | metformin | 857 | 55.20 | 8.91 | - |
| 120 | NCT00798161 2010 24w-2 | 24w | DPP-4i | placebo | metformin | 857 | 55.20 | 8.91 | - |
| 121 | NCT00894322 2015 | 12w | GLP-1 RAs | placebo | metformin+TZD | 35 | 52.46 | - | - |
| 122 | NCT01023581 2013 26w-1-pc | 26w | DPP-4i | placebo | - | 334 | 53.14 | - | 3.96 |
| 123 | NCT01023581 2013 26w-2-pc | 26w | DPP-4i | placebo | metformin | 450 | 53.89 | - | 4.06 |
| 124 | NCT01076075 2011 | 24w | DPP-4i | placebo | metformin+ sulfonylurea | 422 | 54.90 | 8.40 | - |
| 125 | NCT01076088 2015 | 24w | DPP-4i | metformin | metformin | 617 | 52.47 | 8.64 | - |
| 126 | NCT01149421 2015 | 26w | GLP-1 RAs | placebo | - | 755 | 56.48 | - | 8.34 |
| 127 | NCT01183013 2014 | 54w | DPP-4i | placebo | TZD(pioglitazone) | 763 | 57.28 | 8.12 | - |
| 128 | NCT01204294 2012 | 52w | DPP-4i | metformin | sulfonylurea/TZD/ Acarbose | 574 | 60.90 | 7.99 | - |
| 129 | NCT01215097 2013 | 24w | DPP-4i | placebo | metformin | 305 | 55.50 | 7.99 | - |
| 130 | Roden M 2013 | 76w | DPP-4i;  SGLT-2i | placebo | - | 899 | 55.00 | 7.88 | - |
| 131 | NCT01438814 2014 | 14w | DPP-4i | placebo | metformin | 689 | 53.00 | - | - |
| 132 | NCT01590797 2015 | 24w | DPP-4i | placebo | - | 467 | 57.60 | - | - |
| 133 | NCT01648582 2012 | 52w | GLP-1 RAs | Insulin | metformin/  sulfonylurea | 774 | 55.00 | - | - |
| 134 | NCT02002221 2016 | 12w | DPP-4i | placebo | - | 156 | 59.30 | - | - |
| 135 | Nowicki M 2011 | 52w | DPP-4i | placebo | oral antidiabetic  drugs | 170 | 66.50 | 8.30 | 16.65 |
| 136 | Olansky L 2011 | 44w | DPP-4i | placebo | metformin | 1250 | 49.70 | 9.87 | 3.35 |
| 137 | Pan C 2012 | 24w | DPP-4i | placebo | - | 568 | 51.40 | 8.15 | 1.00 |
| 138 | Pfutzner A 2011 | 24w | DPP-4i | placebo | metformin | 1306 | 51.99 | 9.50 | 1.70 |
| 139 | Pratley R 2011 | 78w | GLP-1 RAs | DPP-4 | metformin | 658 | 55.30 | - | 6.20 |
| 140 | Pratley RE 2009 | 26w | DPP-4i | placebo | metformin+TZD  (pioglitazone)/  sulfonylurea+TZD  (pioglitazone) | 493 | 55.40 | 8.00 | 7.60 |
| 141 | Prato S 2011 | 24w | DPP-4i | placebo | oral antidiabetic  drugs | 503 | 55.70 | 8.00 | - |
| 142 | Prato SD 2015 | 52w | SGLT-2i | sulfonylurea | metformin | 814 | 58.40 | 7.72 | - |
| 143 | Raz I 2006 | 24w | DPP-4i | placebo | - | 741 | 54.20 | 8.00 | 4.50 |
| 144 | Raz I 2008 | 18w | DPP-4 | placebo | metformin | 190 | 54.80 | 9.20 | - |
| 145 | Roden M 2013 | 24w | DPP-4i;  SGLT-2i | placebo | - | 899 | 55.00 | - | - |
| 146 | Rosenstock J 2006 | 24w | DPP-4i | placebo | TZD(pioglitazone) | 353 | 56.20 | 8.00 | 6.10 |
| 147 | Rosenstock J 2009 | 24w | DPP-4i | placebo | metformin | 401 | 53.46 | 7.90 | 2.60 |
| 148 | Rosenstock J 2013 | 52w | DPP-4i | sulfonylurea | - | 441 | 69.90 | 7.47 | 6.10 |
| 149 | Rosenstock J 2015 | 24w | DPP-4i | SGLT-2i | metformin | 266 | 54.50 | 8.95 | 7.80 |
| 150 | Rosenstock J 2016 | 26w | GLP-1 RAs | insulin | - | 894 | 59.80 | - | - |
| 151 | Rosenstock J 2018 104w | 104w | SGLT-2i | placebo | metformin | 621 | 56.60 | 8.10 | 8.00 |
| 152 | Rosenstock J 2018 52w | 52w | SGLT-2i | placebo | insulin | 730 | 45.00 | 8.10 | 22.53 |
| 153 | Rosenstock J 2019 | 24w | DPP-4i | SGLT-2i | metformin | 870 | 56.70 | 8.20 | 7.60 |
| 154 | Ross SA 2012 | 12w | DPP-4i | placebo | metformin | 491 | 58.60 | 7.97 | - |
| 155 | SAVOR-TIMI Raz I 2014 | 109w | DPP-4i | placebo | insulin/metformin/  sulfonylurea | 16492 | 65.00 | 8.00 | 10.30 |
| 156 | SCALE Davies MJ 2015 | 68w | GLP-1 RAs | placebo | - | 846 | 54.90 | - | 7.30 |
| 157 | Scherbaum Wa 2008 | 52w | DPP-4i | placebo | - | 306 | 63.10 | 6.75 | 2.60 |
| 158 | Shankar RR 2017 104w | 104w | DPP-4i | sulfonylurea | metformin | 402 | 57.15 | 8.05 | 7.80 |
| 159 | Shankar RR 2017 24w | 24w | DPP-4i | placebo | insulin; metformin | 467 | 57.70 | 8.70 | 11.40 |
| 160 | Sheu W 2015 | 52w | DPP-4i | placebo | insulin | 1261 | 60.00 | 8.60 | - |
| 161 | Softeland E 2017 | 24w | DPP-4i;  SGLT-2i | placebo | - | 332 | 55.20 | - | - |
| 162 | START Terauchi Y 2017 | 52w | DPP-4i | sulfonylurea | - | 272 | 70.50 | 7.48 | - |
| 163 | Takeshita Y 2015 | 12w | GLP-1i | DPP-4 | sitagliptin | 122 | 64.70 | 8.00 | - |
| 164 | Takihata M 2013 | 24w | DPP-4i | TZD | metformin/  sulfonylurea/  metformin+  sulfonylurea | 115 | 60.50 | 7.44 | - |
| 165 | TECOS Green JB 2015 | 156w | DPP-4i | placebo | oral antidiabetic  drugs | 14671 | 65.50 | 7.20 | 11.60 |
| 166 | Terauchi Y 2014 | 12w | GLP-1 RAs | placebo | metformin | 145 | 52.17 | - | 4.60 |
| 167 | Vilsboll T 2010 | 24w | DPP-4i | placebo | insulin | 641 | 57.80 | 8.70 | 12.50 |
| 168 | Wainstein J 2012 | 32w | DPP-4i | TZD | metformin | 517 | 52.30 | 8.90 | 3.24 |
| 169 | Wanner C 2016 | 240w | SGLT-2i | placebo | - | 7020 | 63.10 | - | - |
| 170 | Weber MA 2015 | 12w | SGLT-2i | placebo | insulin | 613 | 55.90 | 8.05 | 7.90 |
| 171 | Weber MA 2016 | 12w | SGLT-2i | placebo | metformin | 449 | 56.50 | 8.05 | 7.50 |
| 172 | Yale JF 2014 | 52w | SGLT-2i | placebo | sulfonylurea | 269 | 68.50 | 8.00 | 16.30 |
| 173 | Yang HK 2015 | 24w | DPP-4i | placebo | - | 108 | 56.20 | 7.14 | 3.59 |
| 174 | Yang W 2011 | 16w | GLP-1 RAs | sulfonylurea | metformin | 928 | 53.30 | - | 7.50 |
| 175 | Yang W 2016 | 24w | SGLT-2i | placebo | metformin | 444 | 53.74 | 8.13 | 4.94 |
| 176 | Yoon KH 2011 | 24w | DPP-4i | placebo | - | 520 | 50.90 | 9.50 | 2.10 |
| 177 | Zhou Z 2018 | 156w | SGLT-2i | placebo | - | 5812 | 64.00 | - | - |
